# Supplementary material for: Polymer stealthing and mucin-1 retargeting for enhanced pharmacokinetics of an oncolytic vaccinia virus
Source: Mol Ther Oncolytics. 2021 Mar 17;21:47–61. doi: 10.1016/j.omto.2021.03.011 (PMC8026752; doi:10.1016/j.omto.2021.03.011)
Supplement: Document S1. Supplemental Materials and methods and Figures S1–S3 [file mmc1.pdf]

OMTO, Volume *21*

Supplemental information

Polymer stealthing and mucin-1 retargeting for enhanced pharmacokinetics of an oncolytic vaccinia virus

Claudia Hill, Megan Grundy, Luca Bau, Sheena Wallington, Joel Balkaran, Victor Ramos, Kerry Fisher, Len Seymour, Constantin Coussios, and Robert Carlisle

Polymer Stealthing and Mucin-1 (MUC1) Retargeting for Enhanced Pharmacokinetics of an Oncolytic Vaccinia Virus

Claudia Hill[1], Megan Grundy[1], Luca Bau[1], Sheena Wallington[1], Joel Balkaran[1], Victor Ramos[2], Kerry Fisher[3], Len Seymour[3], Constantin Coussios[1] and Robert Carlisle[1]

[1] *Institute of Biomedical Engineering, University of Oxford, UK,* [2] *Grup d'Enginyeria de Materials, Institut Quimic de Sarria, Universitat Ramos Llull,* [3] *Department of Oncology, University of Oxford, UK*

This work was performed in Oxford, UK. Correspondence should be addressed to R.C. (robert.carlise@eng.ox.ac.uk)

Short title: Polymer coating and retargeting of oncolytic VV

Keywords: vaccinia virus; oncolytic virotherapy; polymer coating; polymer coating; cholesterol-PEG; targeting; mucin 1; capan-2; pancreatic cancer

Supplementary information:

Methods:

In a further biodistribution study, mice were dosed with $1x10^8$ VG of luciferase expressing VV, PCVV or aMUC1-PCVV (Figure S3). 24 hours after mice were treated with $1x10^8$ VG IVIS imaging was performed to assess the biodistribution and luminescence in livers and tumours. Mice were anaesthetised with 2-3% isoflurane in O2 enhanced air and then were injected intraperitoneally with 100µL of 15.8 mg/mL luciferin (15225733, ThermoFisher). 100µL Pierce™ D-Luciferin, Monopotassium Salt (ThermoFisher Scientific, 88294) was used at 15.8 mg/mL in PBS by intraperitoneal injection. The dose was split between two injections of 50µL on each side of the abdomen of the mouse being imaged. 5 minutes after luciferin administration, imaging was performed using an in vivo imaging system (IVIS Lumina, Caliper Life Sciences).

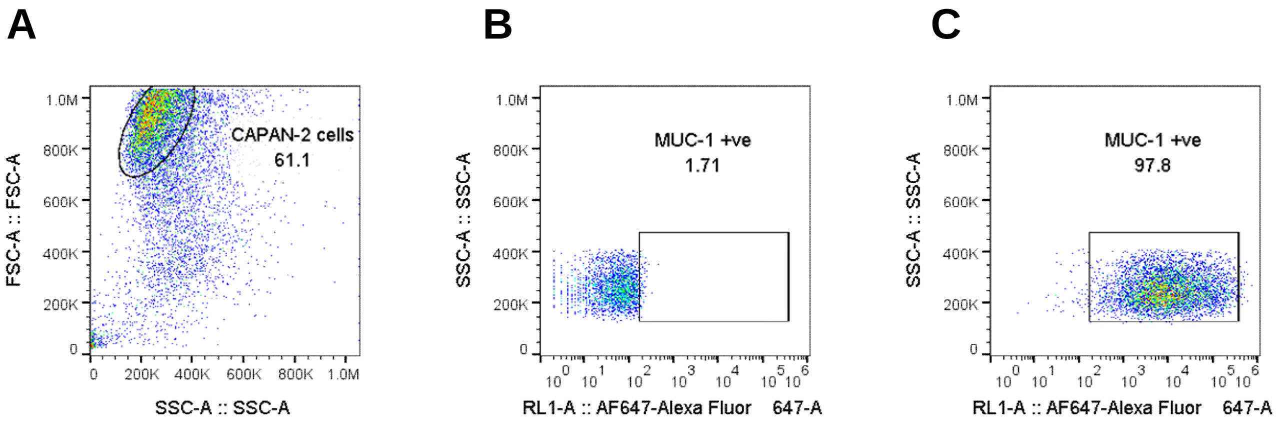

Figure S1 Characterising the binding of aMUC-1 antibodies to a high MUC-1 expressing human cancer cell line CAPAN-2. Flow cytometry was used to determine the binding of aMUC-1 antibodies to CAPAN-2 cells. Cells were harvested and suspended at a $1x10^3$ cells per mL concentration and groups that were stained for MUC-1 were incubated with a 40 µg/ mL concentration of aMUC-1 antibody. Cells were then washed in PBS and aMUC-1 binding was detected using an anti-mouse IgG AF647 secondary. (a) Gating for CAPAN-2 cell population. (b) Gating for positive MUC-1 staining set by unstained CAPAN-2 cells and (c) CAPAN-2 cells stained for MUC-1.

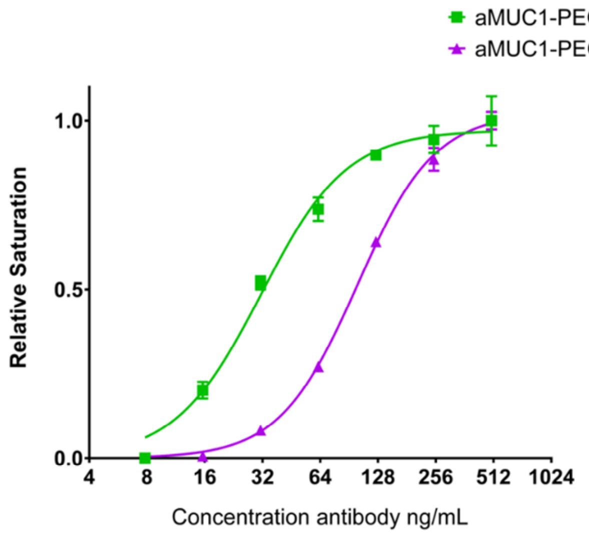

Figure S2 Assessing the impact of incubation of MUC1 peptide on Cholesterol-PEG-aMUC1 binding to immobilised MUC1. An ELISA was performed to compare the binding affinity of aMUC1-PEG-Chol suspended at different concentrations in PBS (green) and aMUC1-PEG-Chol incubated suspended at different concentrations in PBS containing five times the mass of aMUC1 of free MUC1 core peptide (purple) to immobilised MUC1 core peptide on the plate, using an ELISA. Error bars represent SD, n=5.

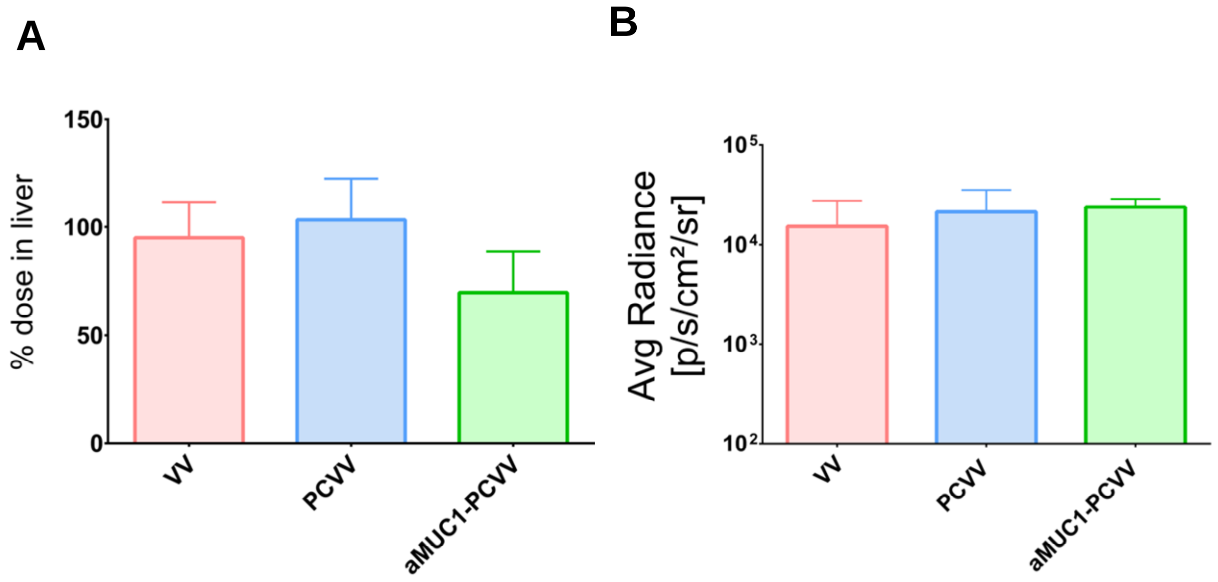

Figure S3 Analysis of livers after dosing with 1x10^{8} VG of VV (pink), PCVV (blue) and aMUC1-PCVV (green). (A) Livers were excised from mice 20 minutes after dosing in PK studies and the % dose of VV, PCVV or aMUC1-PCVV circulating was determined by qPCR for each liver. (B) The mean average radiance was determined in livers 24 hours after dosing. No significant differences were evident between any group in panel A or B. n=4
