# Supplementary material for: Polymer stealthing and mucin-1 retargeting for enhanced pharmacokinetics of an oncolytic vaccinia virus
Source: Mol Ther Oncolytics. 2021 Mar 17;21:47–61. doi: 10.1016/j.omto.2021.03.011 (PMC8026752; doi:10.1016/j.omto.2021.03.011)
Supplement: Document S2. Article plus supplemental information [file mmc2.pdf]

Molecular Therapy
Oncolytics
Original Article

Polymer stealthing and mucin-1 retargeting for enhanced pharmacokinetics of an oncolytic vaccinia virus

Claudia Hill,[1] Megan Grundy,[1] Luca Bau,[1] Sheena Wallington,[1] Joel Balkaran,[1] Victor Ramos,[2] Kerry Fisher,[3] Len Seymour,[3] Constantin Coussios,[1] and Robert Carlisle[1]

[1]Institute of Biomedical Engineering, University of Oxford, Oxford OX3 7DQ, UK; [2]Grup d'Enginyeria de Materials, Institut Quimic de Sarria, Universitat Ramon Llull, Barcelona, Spain; [3]Department of Oncology, University of Oxford, Oxford OX3 7DQ, UK

Vaccinia virus (VV) is a powerful tool for cancer treatment with the potential for tumor tropism, efficient cell-to-cell spread, rapid replication in cancer cells, and stimulation of anti-tumor immunity. It has a well-defined safety profile and is being assessed in late-stage clinical trials. However, VV clinical utility is limited by rapid bloodstream neutralization and poor penetration into tumors. These factors have often restricted its route of delivery to intratumoral or intrahepatic artery injection and may impede repeat dosing. Chemical stealthing improves the pharmacokinetics of non-enveloped viruses, but it has not yet been applied to enveloped viruses such as VV. In the present study, amphiphilic polymer was used to coat VV, leading to reduced binding of a neutralizing anti-VV antibody (81.8% of polymer-coated VV [PCVV] staining positive versus 97.1% of VV [p = 0.0038]). Attachment of anti-mucin-1 (aMUC1) targeting antibody, to give aMUC1-PCVV, enabled binding of the construct to MUC1. In high MUC1 expressing CAPAN-2 cells, infection with PCVV was reduced compared to VV, while infection was restored with aMUC1-PCVV. Pharmacokinetics of aMUC1-PCVV, PCVV, and VV were evaluated. After intravenous (i.v.) injection of 1×10^8 viral genomes (VG) or 5×10^8 VG, circulation time for PCVV and aMUC1-PCVV was increased, with ~5-fold higher circulating dose at 5 min versus VV.

INTRODUCTION

Oncolytic viruses are replication competent viruses that can have a natural or engineered tropism to infect and kill cancer cells.[1] The advantages and limitations of oncolytic viruses have been extensively detailed previously[2] and are exemplified by vectors based on vaccinia virus (VV).

Oncolytic VV has been used to treat hundreds of cancer patients presenting with varying types of cancer in a multitude of late-stage clinical trials to date.[2,3] Pexa-Vec, a thymidine kinase (TK)-deleted VV that encodes granulocyte-macrophage colony-stimulating factor (GM-CSF), has been shown to be safe and effective in a range of cancers, including melanoma,[4,5] liver cancer,[6–11] and colorectal cancer[9,12] Recently, however, the phase III trial (ClinicalTrials.gov: NCT02562755) "PHOCUS" investigating the benefits of the combination of Pexa-Vec + sorafenib compared to sorafenib alone was stopped, as it became clear that the trial would not meet its primary endpoint of overall survival.

Notably, out of the 24 studies reported investigating oncolytic VV clinically, only 8 used intravenous (i.v.) administration, and to date marked improvement in survival has only been shown with intratumoral (i.t.) administration. This may be the reason that most ongoing or recruiting trials[13] are using i.t. administration (ClinicalTrials.gov: NCT02562755, NCT02977156, and NCT03071094) or localized injection. Oncolytic VV delivered i.t. has shown a good safety profile and some promising anti-tumor response. However, achieving effective dissemination throughout the body is still an area that needs improvement if expansion of the clinical utility and ease of administration are to be realized.

It has been shown in pre-clinical models that the bloodstream stability and tumor accumulation of oncolytic viruses can be improved using biocompatible polymers to shield the virus from antibodies and interactions with other blood components. By shielding viruses from pre-existing neutralizing antibodies, their efficacy can be improved. Shielding of viruses can occur via genetic and chemical engineering,[14,15] and it is the latter of these approaches that is the focus of this work.

Chemical coating of the adenovirus (Ad) has been a particularly well-researched field, with poly(ethylene glycol) (PEG),[16] poly(*N*-(2-hydroxypropyl)methacrylamide) (pHPMA),[17] albumin,[18] and gold[19] all having been applied to enhance the pharmacokinetics of Ad vectors. Such strategies have also allowed for the attachment of ligands to the polymer coat to provide specific redirection of the virus tropism.[17,20,21] To date, although genetic and enzymatic modifications of the VV

Received 19 June 2020; accepted 14 March 2021;
https://doi.org/10.1016/j.omto.2021.03.011.

Correspondence: Robert Carlisle, Institute of Biomedical Engineering, University of Oxford, Oxford OX3 7DQ, UK.
E-mail: robert.carlise@eng.ox.ac.uk

This is an open access article under the CC BY license (http://creativecommons.org/licenses/by/4.0/).

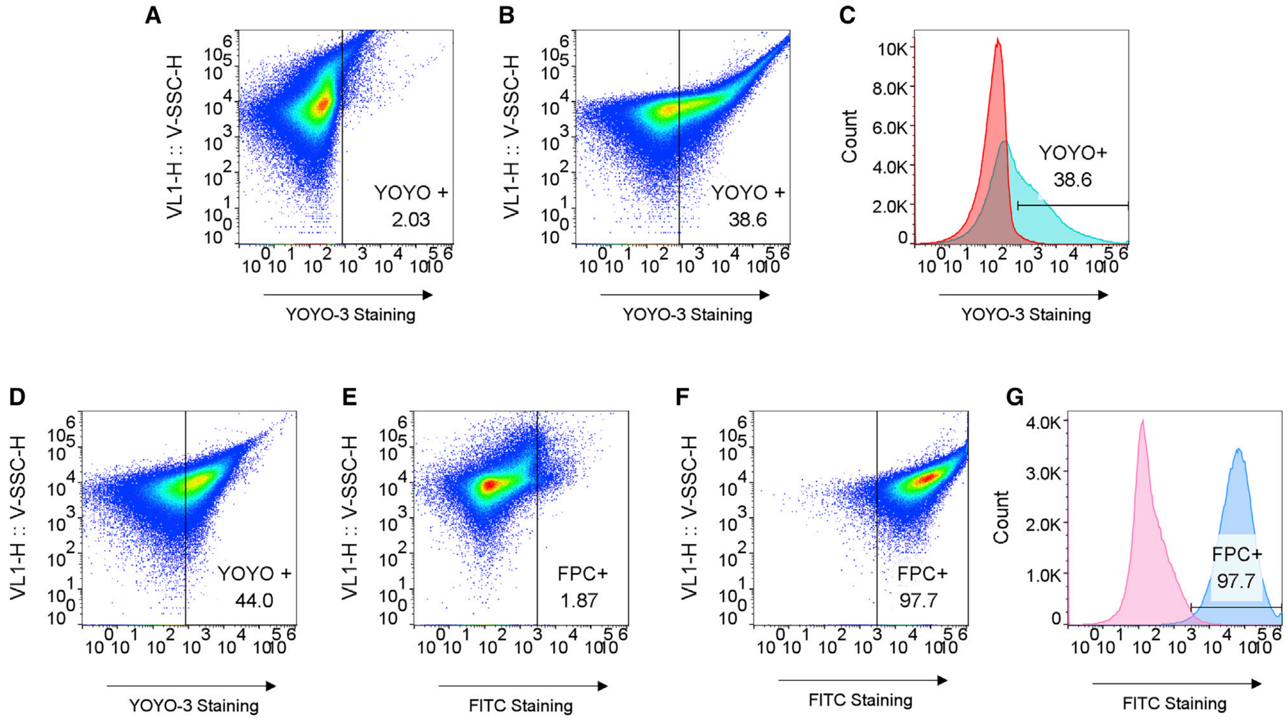

Figure 1. Identifying VV population using flow virometry with a nucleic acid stain (YOYO-3) and confirmation of coating with a FITC-conjugated polymer (FPC)

(A and B) Solutions were at a concentration of 2.6×10^5 viral genomes (VG)/μL: YL2-H versus violet SSC (V-SSC) for VV (A) and VV + YOYO (B), respectively. The events counted were 3.55×10^5 and 4.31×10^5, respectively. (C) Histogram in YL2-H: red-colored peak on the histogram is VV, and blue-colored peak on the histogram is VV + YOYO. (D) YL2-H versus V-SSC for FITC-PCVV + YOYO ungated. (E and F) BL1-H versus V-SSC plots of VV + YOYO (E) and FITC-PCVV + YOYO (F), from a YOYO-3 gated VV population. The events counted were 1.66×10^5 and 1.74×10^5, respectively. (G) Histogram displaying the FITC fluorescence of gated VV (pink, geometric mean fluorescence [FL] = 159) and FITC-PCVV (blue, geometric mean FL = 4.88×10^4) populations. Data are representative of n = 3 repeats on two separate occasions.

surface have been described with varying degrees of success,[22–27] there has been a paucity of attempts to chemically modify VV for enhanced protection and retargeting. Previously it has been shown *in vitro* that genetic retargeting of modified VV Ankara (MVA), a heavily attenuated poxvirus, to mucin-1 (MUC1)-positive cells was possible.[26] Herein, we report the use of a cholesterol-PEG polymer and an anti-MUC1 (aMUC1) antibody to provide such coating and retargeting for an oncolytic VV to cellular MUC1. MUC1 is a tumor-associated antigen with overexpression often associated with cancers, including colon, breast, ovarian, and pancreatic cancer.[28,29]

The oncolytic VV utilized in this study is an attenuated recombinant VV that is derived from the Copenhagen strain, encodes luciferase, and has a deletion of the TK and ribonucleotide reductase genes. This double deleted VV is highly attenuated in normal cells, yet it displays tumor-selective replication and cell kill.[30]

RESULTS

PEG-cholesterol (PEG-Chol) coating of VV confirmed using flow virometry

To test PEG(8 kDa [8K])-Chol coating of VV, a fluorescently labeled PEG-Chol polymer was used to coat VV, and the VV particles preincubated with the fluorescent fluorescein isothiocyanate (FITC)-PEG(10 kDa [10K])-Chol (FPC) were analyzed using an Attune NxT cytometer (see Confirmation of Chol-PEG coating VV using flow virometry). The use of a flow cytometer to analyze viral particles, i.e., flow virometry,[31] has been previously used to identify VV,[32] and so it was hypothesized that this technique could also be used to investigate coating of VV.

Due to high background noise in some initial tests, a nucleotide stain, YOYO-3, was used to enhance the detection of VV using the Attune NxT. Solutions of VV and VV + YOYO-3 were analyzed, and the results are shown in Figure 1.

The population that was observed for VV in forward scatter (FSC) versus side scatter using a violet laser (V-SSC) plots (data not shown) was in line with what is reported in the literature; that is, VV was identifiable in SSC but not in FSC.[31,32] Therefore, plots are shown comparing the V-SSC and YOYO-3 fluorescence. As seen in Figures 1A–1C, a YOYO-3-stained VV population was successfully identified. As YOYO-3 is an orange fluorescent nucleic acid stain, any fluorescent particles detected in the YL2 (561–620/15) channel should correspond to stained VV particles. Figure 2B shows that 38.6% of

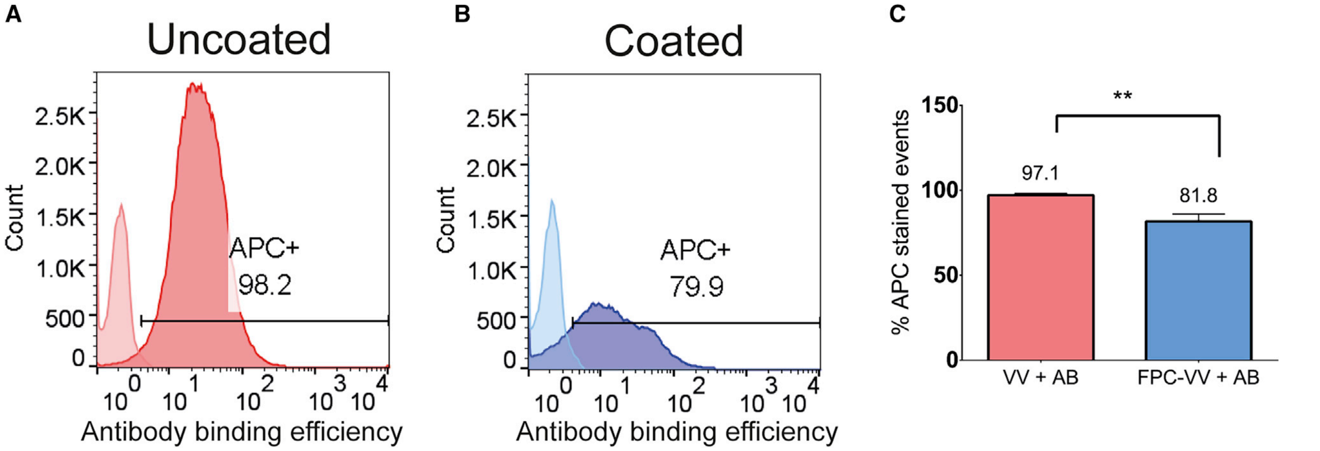

Figure 2. Comparing the binding of a neutralizing anti-vaccinia antibody to coated and uncoated VV samples using flow virometry and a human anti-vaccinia primary antibody (NAb) and APC fluorophore-labeled anti-human secondary antibody

Events with fluorescence above the VV or PCVV without NAb were considered to be positively stained for APC and are denoted APC^+. (A) Representative histogram of the VV + NAb group (red) overlaid on VV without Nab (light red). Naked VV (red) showed that 98.2% of events were positive for APC fluorescence. (B) Representative histogram of the FITC-PCVV + NAb group (blue) overlaid on PCVV without Nab (light blue). (C) Graph displaying the mean percentage of APC-stained events. Error bars represent SD. Statistical significance was determined using an unpaired two-tailed t test. $p = 0.004$; $n = 3$.

the events detected stained positively for YOYO-3. Successful identification of the YOYO-3-stained VV population allowed for VV populations to be determined by gating for YOYO-3 fluorescence in further studies that sought to characterize coating of VV with a FITC-PEG-Chol. FITC-PEG(10K)-Chol was preincubated with VV and washed as described in section 4.1. Flow virometry was used to analyze and compare samples of FITC-PEG(10K)-Chol-VV (FITC-PCVV) and VV alone. The results are shown in Figures 1D–1G.

The recorded events were gated for VV using YOYO-3 fluorescence, and viral FITC fluorescence was used to confirm the polymer coating via flow virometry analysis using the BL1 channel (488–530/30). Virus particles with a fluorescence higher than the control sample (VV alone) were considered coated with a FITC-PEG(10K)-Chol, “FITC-PEG-Chol positive,” and were gated for. The percentage of events that were within this gate were quantified and denoted “FPC^+.”

Comparing the BL1 histograms for VV and FITC-PCVV (Figure 1G), distinct peak fluorescence intensities were observed for each sample. Non-modified VV had a very low fluorescence (geometric mean fluorescence value of 159) when compared with the FITC-PCVV sample (which had a geometric mean fluorescence of 4.9×10^4), suggesting that despite many washes, the FITC-PEG(10K)-Chol was successfully embedded in the VV membrane, and the VV population (97.7% positive) was highly fluorescent.

Similar results were also found on a different flow cytometer (BD FACSCalibur). In these experiments (data not shown), VV populations were gated by size, and fluorescence from a FITC-PEG(10K)-Chol was used to confirm the polymer coating via flow virometry analysis of fluorescence in the FL1 channel. A strong FITC signal was obtained with a mean, across n = 4 replicates, of 99.3% of events showing positive FITC staining and more than a 1,000-fold increase in mean fluorescence intensity when compared with unmodified VV across all samples.

To ensure that the cholesterol group, and not the FITC group, was the element of the FITC-PEG(10K)-Chol chain that was embedding into the VV membrane, a FITC-PEG + VV control was analyzed. The VV population of the FITC-PEG + VV sample had a geometric mean fluorescence of 142 which was 10-fold lower than the mean fluorescence of FITC-PCVV (1,367). The populations of FITC-PCVV and FITC-PEG + VV gated for VV contained a similar number of events, 5,446 and 5,829, respectively. This indicates that although there was a small amount of PEG-FITC attaching to VV membranes, it was substantially less than FITC-PEG(10K)-Chol. This indicates successful coating of VV by FITC-PEG(10K)-Chol, even after washing, via the proposed mechanism of cholesterol embedding in the VV lipid membrane.

PEG-Chol coating reduces VV binding of neutralizing antibodies

One way to extend the circulation half-life of VV is to reduce the binding of anti-VV neutralizing antibodies (NAbs). Therefore, studies were performed to determine the effects of coating VV on antibody binding. In this study, we investigated the binding of a NAb to unmodified VV and PCVV particles, formulated and stained as described in sections 4.1 and 4.1.1, respectively.

Virus particles were incubated for 40 min with a NAb for VV in a virus-to-antibody ratio of 1:50. Binding of the NAb was detected by an anti-rabbit immunoglobulin (Ig)G secondary antibody conjugated to an allophycocyanin (APC) fluorophore. APC fluorescence was used to quantify the antibody binding via flow virometry. Samples were first gated for a VV population as described in section 4.1.2. Figures

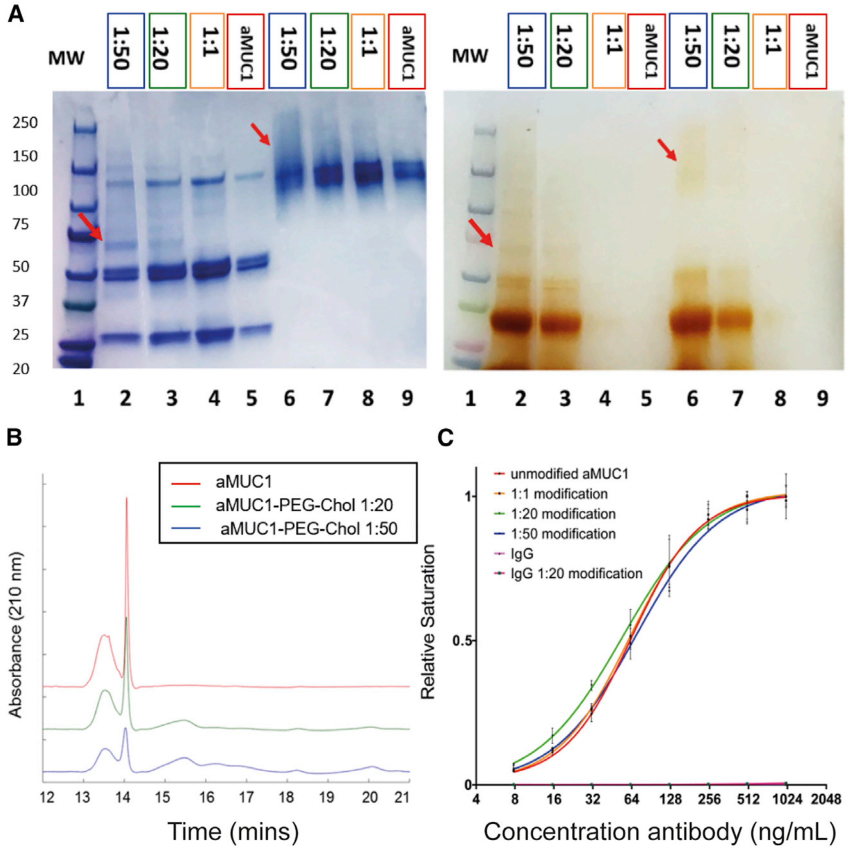

Figure 3. Conjugation of cholesterol (Chol)-PEG-NHS to a MUC1 antibody

(A) Characterization of PEGylated antibodies using SDS-PAGE with Coomassie (left) and barium iodide (right) staining. Samples run in reducing conditions are in lanes 2–5, and samples run in non-reducing conditions are in lanes 6–9. Conjugation success was compared for Chol-PEG-NHS concentrations of 6.67 μM (Ab/polymer ratio of 1:1), 132 μM (1:20), and 333 μM (1:50). (B) HPLC analysis of unmodified aMUC1 (red) and aMUC1 modified with 132 μM (green) or 333 μM Chol-PEG-NHS (blue). Chromatographs shown were acquired at an absorbance of 210 nm and were baseline corrected. (C) MUC1 binding capability of aMUC1 (red) and aMUC1 modified with 6.67 μM (orange), 132 μM (green), or 333 μM Chol-PEG-NHS (blue) using an ELISA. IgG (pink) and IgG modified with 132 μM Chol-PEG-NHS (dark pink) were used as binding controls (neither showed levels above background). Error bars represent SD; n = 5.

2A and 2B show representative histograms of VV and PCVV antibody binding, respectively. Virus particles with a fluorescence higher than the control sample (VV or PCVV alone) were considered APC-positive and were gated for. The percentage of events that were within this gate were quantified and denoted "APC$^+$." The mean percentage of events stained for APC of three samples was taken and the results are shown in Figure 2C. There was significantly higher NAb binding of VV, with a mean of 97.1% events bound with NAb when compared with binding of 81.8% PCVV events (p = 0.004).

Chol-PEG-NHS conjugation to aMUC1 antibody

aMUC1 antibody was modified and conjugated to VV via a Chol-PEG linker. aMUC1 was first conjugated to Chol-PEG-*N*-hydroxysuccinimide (NHS), and sodium dodecyl sulfate polyacrylamide gel electrophoresis (SDS-PAGE) and high-performance liquid chromatography (HPLC) analysis were used to verify successful conjugation. After separation by SDS-PAGE, unmodified and PEGylated aMUC1 antibodies (reacted with Chol-PEG-NHS at varying polymer concentrations) were analyzed using a barium iodide stain for PEG, followed by a Coomassie protein stain. Incubation with barium iodide for 15 min followed by rinsing with pure water showed staining in lanes where the polymer had been loaded (Figure 3A, right panel). The gel was then washed in Milli-Q water until the barium iodide stain had disappeared, and then incubated with a Coomassie stain until the gel was stained uniformly blue. Subsequently, the gel was destained and imaged (Figure 3A, left panel). It has been shown previously that PEGylation affects the apparent molecular weight (MW) of proteins, and that overlap of a Coomassie stain with a barium iodide stain can indicate successful PEGylation of protein.[33] In this study, we observed that non-reduced PEGylated aMUC1 appeared as a smeared band (lanes 6–8) when compared to unmodified non-reduced aMUC1 (lane 9), which appeared as a relatively sharper band at approximately 150 kDa. The degree of "smearing" of the protein band appeared to be dependent on the concentration of Chol-PEG-NHS used for conjugation, as expected when conjugation occurs randomly on lysine side chains.

The barium iodide staining indicated that, despite washing with centrifugal filtration, free un-conjugated polymer was still present in samples that were loaded onto the gel. The amphiphilic Chol-PEGs form micelles that are of similar size to the conjugated antibodies and hence cannot be separated using size exclusion methods.[34,35]

Observation of faint brown bands overlapping with Coomassie-stained protein bands in lanes 2, 3, 6, and 7 indicated successful PEGylation of aMUC1 antibodies (relevant bands have been highlighted with red arrows). In the reduced samples, several distinct bands were observed after Coomassie staining in the samples where antibody was incubated with polymer at a ratio of 1:50 and 1:20, indicating that modification of individual antibodies may have occurred to different extents. Due to the multitude of bands seen in the modified antibody lanes, further analysis using HPLC was undertaken to confirm PEGylation of the 1:20 and 1:50 modified samples. Three traces are presented in Figure 3B: unmodified aMUC1 (red) and aMUC1 incubated with polymer at ratios of 1:20 (green) and 1:50 (blue). Unmodified

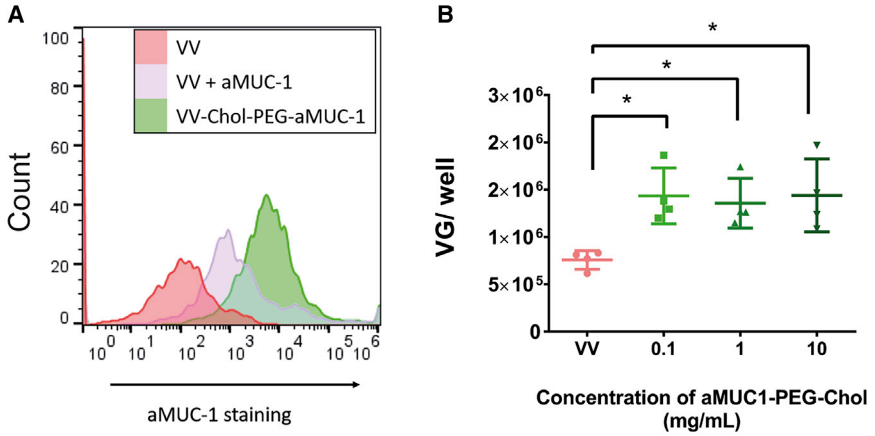

Figure 4. Analysis of aMUC1-PEG-Chol coating VV to form aMUC1-PCVV and assessment of its ability to bind MUC1 target

(A) Histogram for aMUC1 staining to confirm coating with aMUC1-PEG-Chol. Solutions of VV (red), VV + aMUC1 (purple), and VV + aMUC1-PEG-Chol (green) were at a concentration of 6.35×10^5 VG/μL. The events counted were 3.09×10^3, 1.98×10^3, and 2.30×10^3, respectively. Geometric mean fluorescence levels of aMUC1 staining for VV-gated populations of VV, VV + aMUC1, and VV + aMUC1-PEG-Chol were 7.08, 828, and 4,788, respectively. Percentage of VV-gated particles positively stained for AF647 were VV (2.13), VV + aMUC1 (36.7), and VV + aMUC1-PEG-Chol (73.8). (B) The ability of aMUC1-PCVV to bind its MUC-1 target was determined by comparing the binding of naked VV (pink) and aMUC1-PCVV formed at aMUC1-PEG-Chol concentrations of 0.1, 1, and 10 mg/mL (shades of green) to an immobilized MUC1 peptide. After several washing cycles, DNA was extracted from each well and qPCR was performed to determine the quantity of VV DNA present (n = 4). The limit of detection for the qPCR was 200 VG/well. Error bars represent SD. Statistical significance was assessed with an ordinary one-way ANOVA followed by a post hoc Tukey's multiple comparison test. *$p < 0.05$.

aMUC1 consists of two resolved peaks centered around retention times of 13.5 and 14.1 min. The decreased area of the unmodified aMUC1 peaks in the reaction samples, together with the appearance of new broad peaks at 15.5 and 16.5 min, indicates conversion of the starting material. As expected, conversion was concentration-dependent, with a maximum of 75% conversion obtained in the 1:50 sample.

The 16.5 min peak, consistent with more extensive modification, is only detectable in the 1:50 sample. The broadness of the product peaks is consistent with the SDS-PAGE results and is attributable to the MW distribution of the polymer and to the random nature of lysine modification. The combination of the overlapping Coomassie and barium iodide stains and the HPLC analysis provides strong evidence of successful PEGylation of aMUC1. Stability of the aMUC1-PEG-Chol antibody conjugate has been shown by HPLC for both 1:20 and 1:50 modified samples stored at 4°C to be at least 3 months (data not shown).

Once aMUC1 was PEGylated, an indirect ELISA was used to determine the ability of modified aMUC1 to bind its target antigen, a MUC1 core peptide. A MUC1 core peptide was covalently immobilized onto a 96-well plate, and the binding of PEGylated aMUC1 (modified with Ab-to-polymer concentration ratios of 1:1, 1:20, and 1:50) was compared with unmodified aMUC1. The binding curves are shown in Figure 3C. All modified aMUC1s were shown to maintain their ability to bind to the MUC1 target; however, note that for the 1:50 modified aMUC1, the half-maximal saturation concentration was almost 10% higher than that of the unmodified aMUC1 (69.9 and 63.9 ng/mL, respectively), indicating a decrease in functional affinity; however, a one-way ANOVA evaluated differences to not be statistically significant. Notably, IgG (pink) and IgG-PEG-Chol (dark pink) were used as binding controls and showed very low binding affinity to MUC1, and a signal that therefore did not rise above background in Figure 3C. This shows that the binding of the aMUC1-PEG-Chol to the MUC1-coated plates is mediated by the aMUC1-MUC1 interaction.

Incubation of aMUC1 with Chol-PEG-NHS at a 132 μM concentration (1:20) was shown to be effective at producing an aMUC1-PEG-Chol construct while maintaining antibody affinity for its target, and hence this ratio was used to produce aMUC1-PEG-Chol constructs for further VV modification.

Purification of the aMUC1-PEG-Chol from unreacted PEG-Chol via HPLC before use was not possible, and therefore when embedding into VV was performed, free unbound PEG-Chol as well as aMUC1-PEG-Chol constructs were present. The coating of aMUC1-PCVV is therefore likely to contain a mixture of both aMUC1-PEG-Chol and PEG-Chol. In future experiments, if HPLC purification could be implemented, this would likely lead to more effective retargeting.

Attachment of aMUC1 retargeting ligand to VV via PEG-Chol shown using flow virometry

To determine whether the aMUC1-PEG-Chol construct could be successfully embedded into the VV membrane, flow virometry was used as described in section 4.2.3. Populations of VV were gated for using both a YOYO-3 nucleic stain and anti-VV antibody staining. aMUC1-PEG-Chol-VV constructs were prepared using the same method used to prepare PCVV (see Polymer Coating), and flow virometry was performed as described in section 4.2.3. Back-gating enabled a VV population in FSC and V-SSC to be identified. The murine aMUC1 was stained using a secondary anti-mouse antibody conjugated to Alexa Fluor 647 (AF647), and geometric mean fluorescence values were determined. Events were analyzed for aMUC1 presence (i.e., positive staining of aMUC1 with an AF647-tagged anti-mouse antibody) using the RL1-A laser, and sample fluorescence greater than that of the control group was considered aMUC1 positive.

Histograms for aMUC1 fluorescence of VV gated populations of VV alone, VV incubated with unmodified aMUC1, and VV incubated with aMUC1-PEG-Chol samples are displayed in Figure 4A. Distinct mean fluorescence values were seen for VV, VV + aMUC1, and VV + aMUC1-PEG-Chol samples. A gate for aMUC1-positive staining was set using the unstained naked VV sample. As expected, unstained naked VV (red) had a very low geometric mean fluorescence of 7.1. Naked VV stained for AF647 (data not shown) showed low levels of non-specific staining with 28% of VV-gated particles positively stained for AF647 and a geometric mean fluorescence of 350. VV incubated with aMUC1 prior to staining (VV + aMUC1) had 37% positive staining and a geometric mean fluorescence of 830, showing that there is some interaction between unmodified aMUC1 and VV. A 6-fold increase in geometric mean fluorescence was observed for VV + aMUC1-PEG-Chol (mean of 4,800) when compared with VV + aMUC1 (mean of 830), and these samples had 74% positive staining. This suggests that despite extensive washing, aMUC1-PEG-Chol was successfully embedded into the VV membrane.

aMUC1-PCVV successfully binds to MUC1 peptide target

To verify that an aMUC1-PCVV was able to bind to the MUC1 target, an assay was performed in which the numbers of VV and aMUC1-PCVV bound to an immobilized MUC1 core peptide target were quantified using qPCR, as described in section 4.2.3.2. Naked VV and aMUC1-PCVV (formed at different aMUC1 antibody concentrations) were added to the immobilized MUC1 target at a ratio of 3.5×10^6 viral genomes (VG)/well, and their binding to MUC1 was compared. The results are displayed in Figure 4B. Non-specific binding of naked VV resulted in a mean concentration of 8×10^5 ($\pm 1 \times 10^5$) VG/well (equating to 22% ± 3% of the VV copies initially added) being recovered from wells after washing. aMUC1-PCVV samples with polymer coating formulated from polymer containing varying antibody-targeting ligand concentrations of 0.1 ($p = 0.023$), 1 ($p = 0.046$), and 10 mg/mL all showed significantly higher MUC1 binding than did naked VV with 1.4×10^6 (± 3 x10^5) VG/well (41% ± 8% bound), 1.4×10^6 ($\pm 3 \times 10^5$) VG/well (39% ± 7% bound), and 1.4×10^6 (± 4 x10^5) VG/well (41% ± 11% bound), respectively.

It is evident that the assay in Figure 4B is subject to saturation at some point. Studies accompanying Figures 1 and 2 had shown that embedding of polymer was not saturable until 10 mg/mL (data not shown), and so it is thought that the saturation effect in Figure 4B results from an exhaustion of all available MUC1 on the plate.

MUC1-targeted VV infects MUC1-positive cancer cells more readily than PCVV *in vitro*

To determine the ability of a retargeted polymer-coated VV (aMUC1-PCVV) to infect cells expressing high levels of MUC1, CAPAN-2 cells (Figure S1) were incubated in suspension and infected with VV, PCVV, aMUC1-PCVV, or a MUC1 peptide "blocked" MUC1-aMUC1-PCVV. VV used to make up virus samples encodes luciferase, as described in Foloppe et al.[30] Virus samples were made up as described in section 4.1, added to CAPAN-2 cells in suspension at a concentration of 5 VG/cell, and incubated in rotation at 37°C for 2 h. Infected cells were then spun down, resuspended in fresh medium, and added to a 96-well plate in replicates of four. After 24 h, cells were lysed and analyzed for luciferase expression. Luminescence values from individual wells were adjusted for total well protein content (as determined by the Bradford protein assay) and are displayed in Figure 5.

Naked VV showed a high infection capacity at this 0.1 multiplicity of infection (MOI) in the CAPAN-2 cell line, with a mean normalized luminescence of 1.5×10^4 ($\pm 3 \times 10^3$) arbitrary units (a.u.)/g protein. PCVV had a statistically significant ($p = 0.046$) reduced infectivity that resulted in a ~2-fold knockdown in infectivity when compared with that achieved by naked VV, with a normalized luminescence of 7.7×10^3 (± 3 x10^3) a.u./g protein (Figure 5A). However, infectivity of coated VV was restored to approximately that of the naked VV, with the addition of an aMUC1 retargeting ligand resulting in a mean normalized luminescence of 1.4×10^4 ($\pm 5 \times 10^3$) a.u./g protein (Figure 5A). This 2-fold increase in infectivity compared with that of PCVV was found to not be statistically significant ($p = 0.083$).

To determine the specificity of the binding of the aMUC-1 ligand to the MUC1 target, a MUC1 peptide (500 molar equivalents with respect to aMUC1) was used to act in competition with the cellular MUC1 on CAPAN-2 cells for aMUC1-PCVV binding. Initially this was tested by preincubating MUC1 peptide with the aMUC1-PEG-Chol antibody conjugates overnight before they were used to form MUC1-aMUC1-PCVV, as described in section 4.3.2. Preincubation with the MUC1 peptide did reduce the mean infectivity to 9.1×10^3 (± 4 x10^3) a.u./g protein (Figure 5A); however, this difference was not found to be statistically significant ($p = 0.21$) when compared with aMUC1-PCVV 1.4×10^4 ($\pm 5 \times 10^3$) a.u./g protein.

Infectivity of the viral constructs was further tested in the presence of human plasma taken from a donor pre-exposed to vaccinia (Figure 5B). The presence of 2% fresh human plasma greatly impacted the infectivity of all constructs in CAPAN-2 cells. Of all four constructs, naked VV experienced the greatest reduction in its infectivity, showing a 24.5-fold knockdown with a mean normalized luminescence of 6.2×10^2 ($\pm 3 \times 10^2$) a.u./g protein in the presence of 2% human plasma. The modified viruses all showed a lesser reduction of infectivity (PCVV = 1.6-fold, aMUC1-PCVV = 8.9-fold, and MUC1-aMUC1-PCVV = 11.1-fold), perhaps suggesting that the coatings provided some protection against human plasma. PCVV had the lowest knockdown in infectivity with a normalized luminescence of 4.9×10^3 ($\pm 3 \times 10^3$) a.u./g protein, significantly higher than naked VV (6.2×10^2 [$\pm 3 \times 10^2$] a.u./g protein) ($p = 0.020$) and MUC1-aMUC1-PCVV (8.2×10^2 [$\pm 4 \times 10^2$] a.u./g protein) ($p = 0.026$) in the presence of 2% human plasma containing anti-VV antibodies. aMUC1-PCVV had a normalized mean luminescence of 1.6×10^3 ($\pm 1 \times 10^3$) a.u./g protein. Differences in normalized luminescence values between other groups were not shown to be statistically significant.

Further assessment of the specificity of retargeting of aMUC1-PCVV to MUC1 was then performed in which a blocked MUC1-aMUC1-PCVV

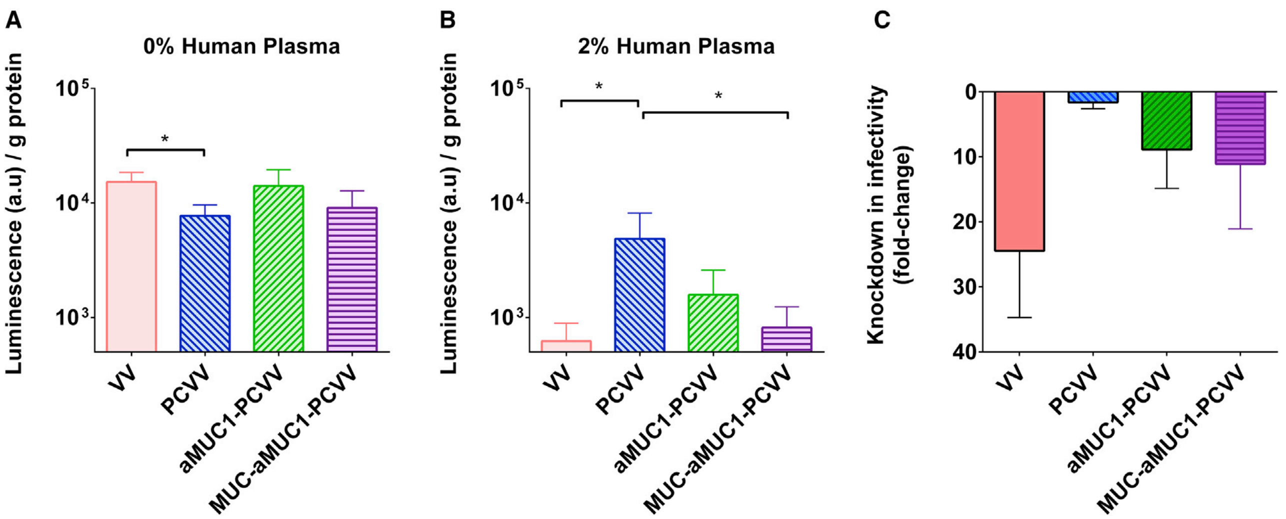

Figure 5. Infection of CAPAN-2 cells after 24 h in the presence or absence of 2% fresh human plasma positive for anti-VV antibodies
CAPAN-2 cells were suspended at a concentration of 1 × 10^5 cells/mL in infection medium containing VV (pink), PCVV (blue), aMUC1-PCVV (green), or MUC1-aMUC1-PCVV (purple) at a final concentration of 5 VG/cell. (A) 0% human plasma. (B) 2% human plasma. (C) Reduction in infectivity due to addition of 2% human plasma. Infection levels were determined by a luciferase assay, and the background luminescence signal from control cells without virus was subtracted. The luminescence values were adjusted by protein content of each replicate well and reported as luminescence per gram of protein. Error bars represent SD. Statistical significance was assessed with an ordinary one-way ANOVA followed by a post hoc Tukey's multiple comparison test. *$p < 0.05$; n = 4.

was formed by incubating the MUC1 peptide (500 molar equivalents with respect to aMUC1) within the infection media at the time of addition of the virus to the cells (as opposed to during the formation of virus coating) (results are shown in Figure 6). Furthermore, the impact of co-incubation with an excess of MUC1 peptide on the ability of aMUC1-PEG-Chol to bind to immobilized MUC1 was shown via an ELISA (Figure S2).

The differences in binding of VV, PCVV, aMUC1-PCVV, or MUC1-aMUC1-PCVV to CAPAN-2 cells was determined. Virus samples were made up as described in section 4.1 and added to cells as described in section 4.3.2. After a 1-h incubation at 37°C, cells were thoroughly washed in phosphate-buffered saline (PBS), DNA was extracted, and VG content was assessed, the results of which are shown in Figure 6A. VV showed the highest level of binding to CAPAN-2 cells with a mean VG per well of 2,260 (±200). The mean VG bound from VV samples was found to be statistically higher than the VG bound from PCVV (1,240 ± 90 VG, $p < 0.0001$), aMUC1-PCVV (1,430 ± 200 VG, $p = 0.0003$), and MUC1- aMUC1-PCVV (700 ± 250 VG, $p < 0.0001$) samples. The addition of the aMUC1 antibody to the coating to form aMUC1-PCVV resulted in an increase in the mean VG bound when compared to that of PCVV; however, this was shown to not be statistically significant ($p = 0.50$). It was shown that the inclusion of blocking MUC1 peptide (MUC1-aMUC1-PCVV) did result in a statistically significant reduction in VG bound when compared with the aMUC1-PCVV sample (2-fold reduction, $p = 0.0009$). This suggested that inclusion of MUC1 peptide in the infection media to compete with cellular MUC1 did indeed impact the cell binding of aMUC1-PCVV to CAPAN-2 cells.

The impact of this reduction in cell binding on infectivity was assessed using the experimental setup described in section 4.3.2. Virus samples were made up as described in section 4.1 and added to CAPAN-2 cells in suspension at a concentration of 5 VG/cell and incubated in rotation at 37°C for 1 h. Cells were then pelleted, resuspended in fresh medium, and added to a 96-well plate. After 24 h, cells were lysed and analyzed for luciferase expression. Luminescence values from individual wells were adjusted for total well protein content, as determined by a Bradford protein assay and are displayed in Figure 6B.

Results were in line with those reported in Figure 5; that is, naked VV had a mean normalized luminescence of 3.50 × 10^5 (±6.74 × 10^4) a.u./g protein, and PCVV had a statistically significant ($p = 0.0268$) reduced infectivity that resulted in an ~2-fold knockdown in infectivity when compared with that achieved by naked VV, with a normalized luminescence of 1.62 × 10^5 (±1.79 × 10^4) a.u./g protein (Figure 5B). Infectivity of coated VV was again restored to that of the naked VV, with the addition of an aMUC1 retargeting ligand resulting in a 3-fold higher mean normalized luminescence of 5.0 × 10^5 (±1 × 10^5) a.u./g protein when compared to that of PCVV ($p = 0.0003$) (Figure 6B). With the MUC1 peptide incubated in the infection media, a 2-fold knockdown in infection was seen with MUC1-aMUC1-PCVV (2.4 × 10^5 [±1 × 10^5] a.u./g protein) when compared with aMUC1-PCVV ($p = 0.003$).

In summary, the data in Figures 5 and 6 suggest that coating VV reduces the impact of human plasma on VV infection of CAPAN-2 cells in these conditions. Furthermore, by introducing an aMUC1 ligand to the coating, this protection is maintained, and overall infectivity is

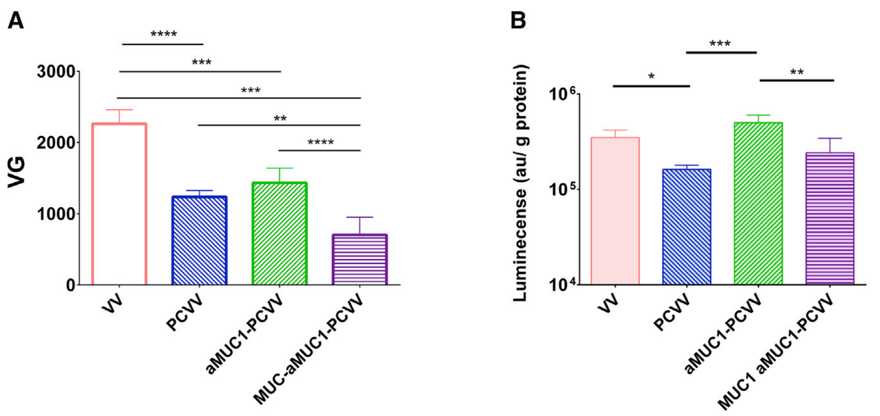

Figure 6. Cell binding after 1-h incubation and infection of CAPAN-2 cells assayed at 24 h

CAPAN-2 cells were suspended at a concentration of 1 × 10^5 cells/mL in infection medium containing VV (pink), PCVV (blue), aMUC1-PCVV (green), or MUC1-aMUC1-PCVV (purple) at a final concentration of 5 VG/cell. MUC1 was incubated at 500 molar equivalents with respect to aMUC1 in the infection media to form MUC1-aMUC1-PCVV (purple). (A) The quantity (VG) of VV, PCVV, aMUC1-PCVV, or MUC1-aMUC1-PCVV bound to cells after 1-h incubation was determined by qPCR. (B) Infection levels were determined by a luciferase assay, and background luminescence signals of cells without virus were subtracted. The luminescence values were adjusted by protein content of each replicate well and reported as luminescence per gram of protein. n = 4; error bars represent SD. Statistical significance was assessed with an ordinary one-way ANOVA followed by a post hoc Tukey's multiple comparison test. *$p < 0.05$, **$p < 0.001$, ***$p < 0.005$, ****$p < 0.0001$.

higher compared to that of the naked VV and a blocked aMUC1-PCVV, indicating selective MUC1-mediated retargeting.

PEG-Chol and aMUC1-PEG-Chol coatings enhance circulation kinetics *in vivo*

Pharmacokinetic (PK) studies were performed to determine the circulation kinetics of VV, PCVV, and aMUC1-PCVV in murine models. BALB/c nude mice bearing subcutaneous CAPAN-2 tumors were treated once tumors reached volumes between 50 and 150 mm^3 as described in section 4.4. Two studies were performed, one at a 1 × 10^8 VG dose and another at a 5 × 10^8 VG dose. Results from both studies are shown in Figure 7A, with the bottom three dashed lines displaying the 1 × 10^8 VG dose-treated mice and the top three lines displaying the 5 × 10^8 VG dose.

For the 1 × 10^8 VG dose, the amount of VV genomes circulating in the mice at 5 min post-injection was statistically significantly increased (approximately 4-fold) for both PCVV (5.7 × 10^6 [±6 × 10^5] VG, p = 0.020) and aMUC1-PCVV (6.0 × 10^6 [± 2 × 10^6] VG, p = 0.014) compared to VV (1.6 × 10^6 [±5 × 10^5] VG). By 10 min, the levels of VV detected in circulation were very low and near the limits of detection. The limit of detection (1.1 × 10^6 VG) is represented by a black dashed line in Figure 7A. This was also true for all three groups at 20 min. Area under the curve (AUC) analysis with a baseline set at the limit of detection showed that exposure was increased for both PCVV (AUC_{0-20} = 1.3 × 10^8 VG min/mL) and aMUC1-PCVV (AUC_{0-20} = 1.3 × 10^8 VG min/mL) compared with VV (AUC_{0-20} = 1.0 × 10^8 VG min/mL).

Circulation kinetics of the virus constructs (PCVV, aMUC1-PCVV, and naked VV) at a higher dose, 5.0 × 10^8 VG, were then investigated. There were no immediate adverse effects seen in the mice at this higher dose. There was a higher quantity of VG circulating for both PCVV (4.0 × 10^7 [±8 × 10^6] VG) and aMUC1-PCVV (6.1 × 10^7 [±2 × 10^7] VG, p = 0.046) at 5 min when compared with VV (2.1 × 10^7 [±1 × 10^6] VG). AUC analysis was also performed on curves at this higher dose with the baseline set at the limit of detection. Once again this showed increased exposure of PCVV (AUC_{0-20} = 9.6 × 10^8 VG min/mL) and aMUC1-PCVV (AUC_{0-20} = 1.1 × 10^9 VG min/mL) compared with VV (AUC_{0-20} = 9.1 × 10^8 VG min/mL). This suggests that the impact of polymer coating on the circulation kinetics of VV was maintained even at a higher dose.

qPCR analysis of tumors recovered 20 min after injection showed very low passive tumor accumulation across all groups dosed at 1 × 10^8 VG (Figure 7B). The mean quantities of VV genome copies recovered in tumors were 8.7 × 10^3 (±6.1 × 10^3) VG/tumor of naked VV, 6.8 × 10^3 (±3.7 × 10^3) VG/tumor of PCVV, and 1.8 × 10^4 (±2.7 × 10^4) VG/tumor of aMUC1-PCVV; however, large variation within groups resulted in no statistical significant difference between groups. Of note, the two highest VG/tumor values were in mice treated with aMUC1-PCVV. We also suspect that given these low values, detection limits were encountered in determining the quantity of tumoral VV. These i.t. viral loads suggest that dissemination into this CAPAN-2 xenograft tumor model was a challenge at a lower 1 × 10^8 VG dose, even for a retargeted virus with an extended circulation time.

qPCR analysis of livers recovered from mice showed rapid clearance of the VV and PCVV from the circulation, in accordance with other studies reported previously.[36] The VV- and PCVV-dosed mice show almost complete sequestration by the liver within 20 min of dosing. In contrast, aMUC1-PCVV-dosed mice showed a substantially reduced (although not significantly different, p = 0.1) level of liver accumulation (Figure S3A). Further analysis of transgene expression of the virus at 24 h was performed to determine whether this lowered liver capture in aMUC1-PCVV-dosed mice impacted the biodistribution and expression in the liver. Analysis demonstrated substantial and equivalent liver expression in all mice regardless of whether they were dosed with VV, PCVV, or aMUC1-PCVV (Figure S3B). Tumor expression was also assessed at 24 h and found to be not substantially

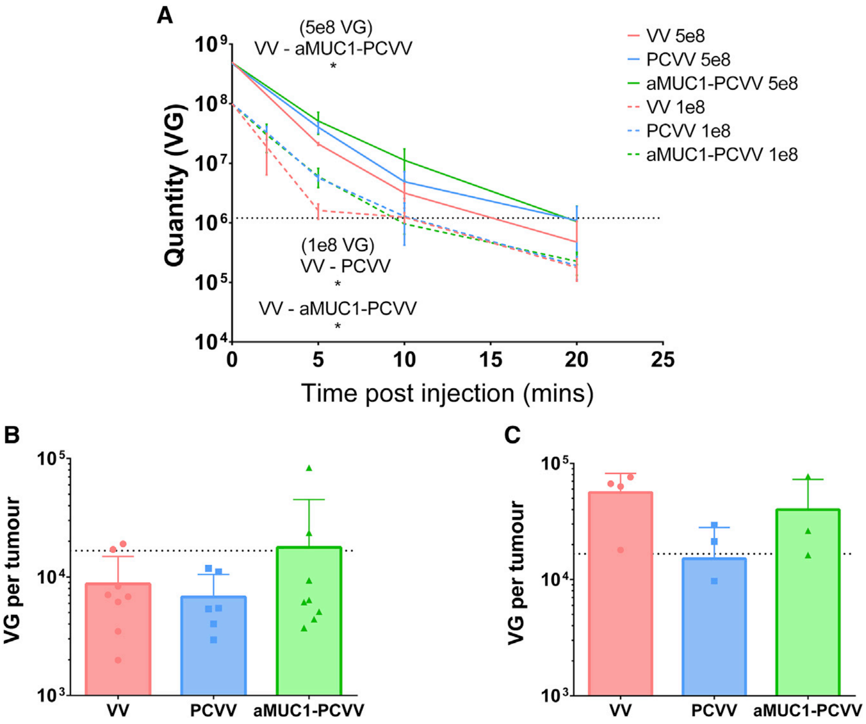

Figure 7. Pharmacokinetic (PK) studies showed that polymer coating and polymer coating with aMUC1 retargeting ligands enhance VV circulation kinetics in mice bearing CAPAN-2 xenografts

(A) PK studies were performed at two doses: 1×10^8 VG (bottom three dashed lines) and 5×10^8 VG (top three lines). The quantity (VG) of a 1×10^8 VG dose and 5×10^8 VG dose of VV, PCVV, or aMUC1-PCVV remaining in circulation was determined by qPCR. In the 1×10^8 VG dose PK study, bleeds were taken from mice at either 2 and 10 min (n = 4) or 5 and 20 min (n = 4). In the 5×10^8 VG dose PK study, consecutive bleeds were taken at 5, 10, and 20 min (n = 4). Error bars represent SD. Ordinary one-way ANOVA was performed with a post hoc Tukey's multiple comparison test. *$p < 0.05$. (B) 1×10^8 VG dose tumors (n = 8). (C) 5×10^8 VG tumors (VV and PCVV, n = 4; aMUC1-PCVV, n = 3). Tumors were resected at 20 min for all mice. After homogenization to 75 mg/mL, DNA was extracted and quantified by qPCR to determine accumulation after 20 min. Results displayed are for the mean VG in each tumor, with error bars representing SD. Limits of detection for qPCR assays are displayed as black dotted lines. Statistical significance was assessed with an ordinary one-way ANOVA. No significance was found for either dose.

above background for any group, in accordance with the low level of accumulation measured after treatment (see Figure 7B). No substantial expression was seen in any other organ.

At a 5×10^8 VG dose, accumulation into tumors was improved (approximately 10-fold greater) for all groups as shown by qPCR analysis on tumors (Figure 7C). The mean quantity of VV genome copies recovered in tumors was 5.6×10^4 ($\pm 2.6 \times 10^4$) VG/tumor of naked VV, 1.5×10^4 ($\pm 1.3 \times 10^4$) VG/tumor of PCVV, and 4.0×10^4 ($\pm 3.3 \times 10^4$) VG/tumor of aMUC1-PCVV. These differences between groups were not found to be statistically significant. Once again, the highest level of VG/tumor was recovered from a tumor (7.7×10^4 VG/tumor) in a mouse treated with aMUC1-PCVV.

DISCUSSION

Oncolytic VV has shown promise as an effective anti-tumor agent in clinical trials, but its efficacy after i.v. administration has been limited by bloodstream neutralization. The scale of this limitation is exacerbated by the fact that a high percentage of the cancer patient population has a baseline anti-VV titer. as VV was used as the vector in the global smallpox eradication vaccine program. Hence, in order for VV to progress into clinical use in a broad patient population diagnosed with a range of cancer types, improving its bloodstream stability is paramount.

In this study, we have shown using flow virometry that chemical modification of VV with PEG(8K) via a cholesterol anchor is possible and is effective at significantly reducing the binding of an anti-VV neutralizing antibody. It is noted that the protection against anti-VV binding reported herein was lower than that which has been previously reported in the literature for polymer-coated Ad. We hypothesize that this is predominantly due to the difference in the polymers used in polymer coating studies of Ad compared to the Chol-PEG polymer used in the work reported herein. First, in Ad studies, polymers were directly attached to the viral capsid via covalent conjugation, whereas in the present study cholesterol was used as an anchor in the lipid membrane of VV. Direct covalent conjugation may result in more efficient polymer coating, but it may also have greater impact on the infectivity of the coated virus than that seen in the studies presented herein. Second, multivalent pHPMA polymers have been shown to provide more effective coating of Ad when compared with PEG, and so it would be worth assessing the use of a Chol-pHPMA polymer coating of VV.[37] Finally, perhaps a combination of both enzymatic glycosylation of VV's surface prior to polymer coating with either a Chol-PEG or Chol-pHPMA could render greater protection of VV from antibody binding and result in an even greater improvement in the circulation profile.[24]

Despite the scope for improvement of coating, the polymer coating of VV did provide a platform onto which we were able to introduce targeting antibodies against MUC1. This was achieved by conjugating a commercial Chol-PEG-NHS heterobifunctional polymer to aMUC1 antibodies via an NHS linker. The cholesterol group on the targeting polymers was then able to embed into the lipid membrane on VV. By conjugating the polymer to the targeting antibody first, we hypothesized that this technique could be used to easily enable retargeting of VV to a number of targeting proteins. Given that the polymer-

antibody structure has also shown stability by HPLC for at least 3 months when stored at 4°C, this may provide an easy, low-cost method of adapting VV treatments to specifically target proteins found to be overexpressed in different cancers.

Tests *in vitro* have shown that this retargeted VV (aMUC1-PCVV) was able to efficiently bind an immobilized MUC1 target. A significant ~2-fold knockdown in infection of CAPAN-2 cells, a cell line shown to have a high expression of MUC1,[38] was achieved when VV was polymer coated (PCVV). Knockdown in infectivity has previously been used to assess the extent to which an oncolytic Ad has been "detargeted" using polymer coating and, hence, observation of a knockdown in infectivity here for VV is in line with those reported for Ad.[39–42] Moreover, this knockdown was then reversed when the aMUC1 targeting ligand was added to the polymer coating. When a MUC1 peptide was incubated with aMUC1-PCVV to form MUC1-aMUC1-PCVV, a limited (non-statistically significant) reduction in mean normalized infectivity was observed. It was further shown that greater infection inhibition was achieved for the MUC1 peptide-blocked control when the MUC1 core peptide was in the infection media rather than premixed with the virus. In the presence of 2% human plasma (containing anti-VV neutralizing antibodies), the three coated viruses showed substantially less reduction in infectivity (PCVV = 2.3-fold, aMUC1-PCVV = 8.9-fold, and MUC1-aMUC1-PCVV = 14-fold) when compared to the naked VV (25-fold). Both PCVV and aMUC1-PCVV had the greatest improved stability in the presence of human plasma, suggesting that the addition of aMUC1 ligands on the coating did not impact the protection that the polymer coating provided to the virus.

In vivo tests were performed to determine the effect of coating with PEG-Chol and aMUC1-PEG-Chol on the circulation kinetics of VV in female BALB/c nude mice. It was shown that mice i.v. injected with 1×10^8 VG of PCVV and aMUC1-PCVV had increased exposure when compared to naked VV. This effect led to a 3.6-fold (PCVV) and 3.7-fold (aMUC1-PCVV) increase in the number of injected VV remaining in circulation 5 min after injection. The enhanced circulation kinetics seen with the PCVV and aMUC1-PCVV groups were also observed at a higher dose of 5×10^8 VG, and no immediate adverse toxic effects were seen in the mice. The immunocompromised BALB/c nude mouse model used in this pharmacokinetic study retains an innate immune response. Hence, the enhanced circulation seen here for aMUC1-PCVV and PCVV suggests that both PCVV and aMUC1-PCVV are able to evade the innate immune response more efficiently than naked VV. This may be due to a lower level of opsonization and better evasion of the mononuclear phagocytic system and hence slower sequestration of VV from the bloodstream to the liver.

It is notable that the improvements evident in the present study do not match the scale of those achieved in studies with non-enveloped viruses. This likely reflects the need to optimize the density of coating on the VV surface, perhaps with the use of multivalent rather than monovalent coating. When considering the increase in circulation time reported herein, it is also important to acknowledge the key role that the adaptive immune system (which is not present in this murine xenograft model) plays in VV clearance, including specifically the role that the anti-VV antibodies play.[43] Our *in vitro* data indicate that polymer coating may result in reduced antibody binding, and this effect is not accounted for in this VV naive mouse model. This increase in bloodstream stability could potentially allow for improved i.v. administration of VV clinically, and studies assessing passive immunization by co-administration with plasma containing anti-VV antibodies would enable this to be determined.

Accumulation of virus in CAPAN-2 xenograft tumors after i.v. injection of 1×10^8 VG was very low across all groups, and there was large variability within groups, potentially due to qPCR assay detection limitations. It is noted that the two highest values for tumoral VV were both in the aMUC1-PCVV group. An ~10-fold increase in tumoral VV accumulation at 20 min in CAPAN-2 xenografts across all groups given a 5×10^8 VG dose was observed compared to tumoral concentrations achieved with a dose of 1×10^8 VG, with the highest viral load detected in a tumor treated with aMUC1-PCVV.

Nevertheless, in this CAPAN-2 cell line, with its dense stromal tissue, it may be that tumoral accumulation despite enhanced circulation half-life is a challenge. We hypothesize therefore that the combination of a retargeted VV with an enhanced circulation half-life with a non-invasive mechanical stimulus, such as focused ultrasound, could potentially lead to improved accumulation within tumors.[37] Indeed, further analysis of retargeting VV to other tumor antigen targets in other tumor models would be an important step in characterizing the amenability of this technique to be applied generally. We endeavor to test these hypotheses in future work.

MATERIALS AND METHODS

Polymer coating

Polymers used for coating of VV in this research were: PEG of MW 8 kDa conjugated at one end to a cholesterol group (*O*-methyl-*O*′-[*N*-(cholesterylsuccinyl)aminoethyl]PEG) 8 KDa, named here as Chol-PEG(8K) (synthesized by VR); PEG of length 10 kDa with a cholesterol group conjugated to one end and a fluorescein conjugated to the other end, (*O*-(fluorescein-5-yl-thioureidoethyl)-*O*′-[*N*-(cholesterylsuccinyl)aminoethyl]PEG 10 KDa), named here as Chol-PEG(10K)-FITC) (Nanocs, PG2-CSFC-10K); and a PEG of length 10 kDa with a cholesterol group conjugated to one end and an NHS group conjugated to the other end (*O*-(succinimidyl carbonate)-*O*'-[*N*-(cholesterylsuccinyl)aminoethyl]PEG 10 kDa), named here as Chol-PEG(10K)-NHS) (Creative PEGWorks, PLS-9985). Differences in the mean length of the PEG chain (8 and 10 kDa) were due to what was commercially available at the time of ordering.

Polymer stock solutions were made fresh before they were mixed with VV. For coating, a 20 mg/mL stock solution of Chol-PEG(8K) was made up in PBS.

VV encoding GFP or luciferase was provided by Transgene SA as frozen stocks with a VG content of 50 per plaque-forming unit

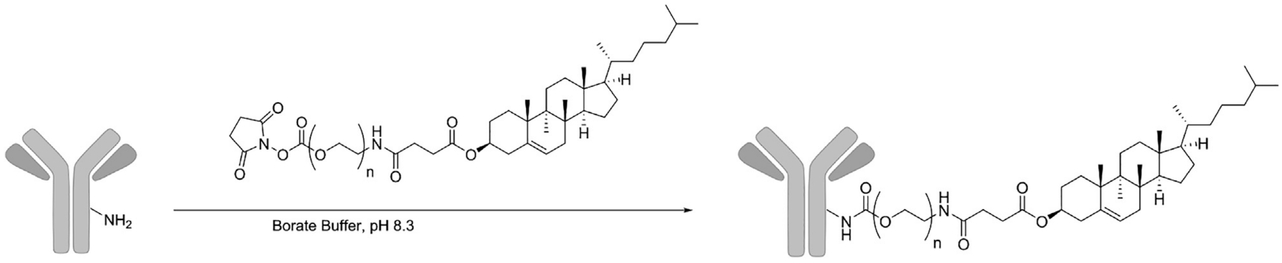

Figure 8. Conjugation of aMUC1 to the coating polymer

(PFU). Coating was achieved by incubation of polymer solution at 10 mg/mL PEG with VV solution at 5 × 10^7 VG/μL. The total volume changed depending on the experiment. This mixture was left at 37°C for 1 h.

The mixture was then diluted 1:10 in Dulbecco's PBS (DPBS) (Sigma-Aldrich, D8537), centrifuged at 20,000 × *g* to pellet Chol-PEG-VV (PCVV), and the supernatant was removed. The process was repeated three times and the final pellet was resuspended in PBS. DNA extraction and qPCR was performed as in Myers et al.[36] to determine viral concentrations. Virus solutions were then resuspended at a final concentration as necessary for the specific assay performed.

Confirmation of Chol-PEG coating VV using flow virometry

To determine whether coating of VV was successful, flow virometry of VV coated with Chol-PEG(10K)-FITC (named FITC-PCVV) was performed.

YOYO-3 (Thermo Fisher Scientific, Y3606) was used for nucleic acid staining of VV.[13,44] VV was incubated with 1 μM YOYO-3 at room temperature for 20 min. The sample was then centrifuged at 20,000 × *g* for 10 min. The viral pellet was washed three times in PBS as described in section 4.1. Final virus pellets were either resuspended in PBS to a concentration of 5.3 × 10^5 virus particles (VG)/μL and further modified to form PCVV, or 2.6 × 10^5 VG/μL as the naked form.[44] Stained VV virions further modified to PCVV were coated and purified as detailed in section 4.1, and resuspended at a final concentration of 2.6 × 10^5 VG/μL. An Attune NxT cytometer (Invitrogen) was adapted to record FSC and SSC through the violet channel using the Attune NxT No-Wash No-Lyse filter kit (Thermo Fisher Scientific, 100022776). VV populations were gated for positive YOYO-3 staining. Analysis was performed using FlowJo v10.6.1 software. FITC-PCVV-YOYO-3 and VV-YOYO-3 were gated for positive YOYO-3 and FITC staining.

Evaluation of the impact of PCVV on antibody binding when compared to VV

Anti-VV antibody binding was determined using flow virometry. VV and PCVV (formulated as described in section 4.1) were made up to a concentration of 1.70 × 10^6 VG/μL. They were then incubated with an anti-VV antibody (Abcam, ab35219) at a 1:50 virus-to-antibody ratio for 40 min at 4°C. Samples were washed and pelleted as describe in section 4.1. They were then incubated with an anti-rabbit antibody conjugated to APC (R&D Systems, F0111) at a concentration of 10 μL per 5 × 10^7 VG for a further 30 min at room temperature. Samples were then washed and resuspended at a concentration 1.70 × 10^6 VG/μL in a total volume of 250 μL. A BD FACSCalibur flow cytometer was used, and analysis was performed using FlowJo v10.6.1 software. VV populations were gated by size.

Retargeting VV

Chol-PEG(10K)-NHS reaction with amine groups of aMUC1 antibody

A polymer with a PEG backbone conjugated to a cholesterol group on one end and an NHS group on the other end (Chol-PEG(10K)-NHS) was used to form aMUC1-polymer conjugates. The NHS group was reacted with primary amine groups on the aMUC1 antibody (Abcam, ab245693) to form an amide bond. This is depicted in the schematic in Figure 8. The reaction took place at polymer concentrations of 330, 132, and 6.67 μM with an antibody concentration of 6.67 μM in a 100 mM borate buffer (pH of 8.3).[45] The polymer-antibody mixture was left to incubate for 60 min, as it was determined by sequential HPLC runs during a 160-min period that the reaction reached completion within 45 min at these conditions. The reaction was quenched with 0.5 M Tris-HCl and finally buffer exchanged into PBS using an Amicon Pro purification system and a 30-kDa filter (Millipore, ACS503012). Successful attachment was then verified using HPLC and SDS-PAGE.

HPLC analysis of PEGylated aMUC1

Samples were analyzed by reverse phase HPLC with an Agilent 1290 Infinity II with a quaternary pump (G7104A) and a diode array detector (G7117B) on a MAbPac RP column (2.1 × 100 mm, 4-μm particle size, 1,500 Å pore size). Chromatographic separation was accomplished with a mobile phase comprised of 0.1% trifluoroacetic acid in water (A) and 0.1% trifluoroacetic acid in acetonitrile (B), with an isocratic hold at 10% B for 4 min followed by a linear gradient from 10% to 90% B during 24 min at a flow rate of 0.35 mL/min and column temperature of 80°C, an injection volume of 8 μL, and UV-Vis detection at 210 and 280 nm.

SDS-PAGE

Samples were mixed with Laemmli buffer (Bio-Rad, 1610747) or reduced in 10% 2-mercaptotheanol (BME, Sigma, M6250) in

Laemmli buffer at a 3:1 ratio and then run on a 4%–20% precast polyacrylamide gel (Bio-Rad, 4561096). Reduced samples were heated at 95°C for 5 min. 10 μL of each sample was loaded into the gel and run at 160 V for 40 min in 1× Tris-glycine-SDS (TGS) running buffer (Bio-Rad, 1610732). Precision Plus Protein Kaleidoscope prestained protein standard (Bio-Rad, 1610375) was loaded to well number 1 for MW analysis.

The presence of PEG was determined by incubating the gels in a barium iodide stain. 5% barium chloride in 1 M HCl was added to a 0.1 N iodine solution at a 5:2 ratio.[33]

The presence of protein was determined by incubating the gels in Coomassie blue stain (0.05 g of Coomassie brilliant blue R dye [Sigma, B7920], 45 mL of methanol, 10 mL of acetic acid, and 45 mL of water) for 2 h and then destaining overnight in a solution of 50% methanol, 10% acetic acid, and 40% water.

ELISA analysis of PEGylated aMUC1

The MUC1 core peptide GVTSAPDTRPAPGSTA (Cambridge Bioscience, ANA60600-1) was diluted to a concentration of 10 μg/mL in 0.05 M (pH 9.6) bicarbonate buffer (Sigma-Aldrich, C3041-50), and 100 μL of this solution was added per well to a 96-well Pierce maleic anhydride-activated plate (Life Technologies, 15110). Plates were incubated at 4°C overnight. Wells were then washed using a PBS and 0.05% Tween 20 buffer and blocked using 10% fetal bovine serum (FBS) in PBS. Unmodified and modified aMUC1 samples were serially diluted to produce a range of antibody concentrations from 0.05 to 6.67 nM. 50 μL of each concentration was added to wells in duplicate for the unmodified and modified aMUC1 samples. Anti-rabbit horseradish peroxidase (HRP)-conjugated antibody (Abcam, ab205718) was diluted 1:5,000 in 10% FBS and 100 μL was then added to each well and incubated for 1 h. Plates were then washed as above and then each well was incubated with 100 μL of tetramethylbenzidine (TMB) substrate (Thermo Fisher Scientific, 34029). Development was stopped by addition of 100 μL of 0.5 M sulfuric acid. Absorbance was read at 450 nm using a plate reader (FLUOstar Omega, BMG Labtech).

Confirmation of Chol-PEG-aMUC1 coating VV

To determine whether coating of VV with the retargeting polymer was successful, flow virometry of VV coated with Chol-PEG(10K)-aMUC1 (aMUC1-PC) was performed. Further qPCR analysis on the binding ability of aMUC1-PCVV to an immobilized MUC1 peptide was also performed.

Flow virometry

The samples were stained for aMUC1 using a goat anti-mouse AF647 secondary antibody (Invitrogen, A-21235). VV populations were determined using a YOYO-3 nucleic acid stain (Thermo Fisher Scientific, Y3606) and an anti-VV antibody (Abcam, ab35219). Preparation and washing were performed as described in section 4.1, with VV incubated for 1 h in excess aMUC1-PEG-Chol (10 mg/mL) solutions and washed vigorously. Flow virometry was performed at a final concentration of 6.35×10^4 VG/μL. An Attune NxT cytometer (Invitrogen) was adapted to record FSC and SSC through the violet channel using the Attune NxT No-Wash No-Lyse filter kit (Thermo Fisher Scientific, 100022776). Analysis was performed using FlowJo v10.6.1 software.

qPCR of VV bound to immobilized MUC1 target peptide

The MUC1 core peptide was immobilized on a Pierce maleic anhydride-activated 96-well plate (Life Technologies, 15110) and blocked as described in section 4.2.2. The final wash step and an additional 10-min rinse before samples were added to the plate was done with PBS (no Tween 20), to avoid lysis of VV.

VV and a range of aMUC1 coating concentrations (0.1, 1, and 10 mg/mL) of aMUC1-PCVV were tested and samples were made up as described in sections 4.1 and 4.2.3.1. For the 0.1 and 1 mg/mL concentration aMUC1-PCVV samples, Chol-PEG was added to make the total concentration of polymer (whether with aMUC1 ligands or without) equal to 10 mg/mL in all three groups, in order to test the effect of the surface density of antibody independently from the surface density of the polymer coating. 3.5×10^6 VG were added to each well in a final volume of 100 μL. Plates were then incubated for 3 h on a shaker plate at room temperature. Samples were then removed from the plate and five washes in PBS were performed with the final wash left for 10 min. 10 μL of proteinase K and 100 μL of cell lysis buffer from the GenElute mammalian genomic DNA miniprep kit protocol (Sigma-Aldrich, G1N350) were then added to each well and left for 30 min on a shaker plate. The contents of each well were then transferred into Eppendorf tubes, vortexed, and incubated at 55°C for 10 min for DNA extraction, and qPCR was performed as in Meyers et al.[36]

Cell work

Cells and culture. Cell work was carried out in a class II microbiological safety cabinet. The medium for cell growth (McCoy's 5A [Thermo Fisher Scientific, 26600023] with 10% FCS [Thermo Scientific, 10270106]), DPBS, and trypsin (1×) (Thermo Fisher Scientific, 15400054) were preheated to 37°C before use.

CAPAN-2 cells (ATCC, HTB-80) were incubated in cell culture flasks at 37°C in 5% CO_2 and split once they reached ~80% confluence. Old growth medium was removed from the flasks and a wash was performed with 10 mL of DPBS. 3 mL of trypsin was then added to the flask and left for 5 min. Once cells had detached from the surface of the flask and were rounded, the trypsin was neutralized by the addition of 10 mL of McCoy's 5A medium flushed over the surface of the flask several times. The cell and medium mixture was transferred to a conical tube and then centrifuged at 181 × *g* for 5 min. After the cells were centrifuged, the medium was removed and replaced with 10 mL of fresh growth medium. The cells were then split into a new flask with fresh growth medium in a 1:4 ratio or counted and used for infection experiments.

A Neubauer chamber was used to determine the concentration of cells suspended in a solution. 100 μL of PBS, 50 μL of trypan blue solution (Thermo Fisher Scientific, 15250061), and 50 μL of the cell solution

being counted were combined and added to the Neubauer hemocytometer. Cells were counted under the microscope.

Infection and cell binding

CAPAN-2 cells were harvested and counted as described in section 4.3.1 and diluted to 2×10^5 cells/mL in McCoy's 5A growth medium. Virus solutions were made up as described in section 4.1 using VV encoding luciferase (detailed in section 1). VV and modified VV samples were added to cells to a final 0.1 MOI and 1 $x10^5$ cell/mL. In studies using human plasma (from a donor with known prior exposure to VV due to vaccinia vaccination), the plasma underwent one freeze-thaw cycle before use and was added in solution to cells at the same time as the VV and modified VV samples. Blocked aMUC1-PCVV samples (MUC1-aMUC1-PCVV) were made by incubating aMUC1 antibodies with 5 times the mass of aMUC1 antibodies of MUC1 peptide overnight at 4°C before being incubated with VV. Solutions were incubated at 37°C on a rotor wheel for 2 h to allow for viral infection. In the second infection study reported herein, blocked aMUC1-PCVV samples (MUC1-aMUC1-PCVV) were made by adding 500 molar equivalents, with respect to aMUC1, of MUC1 peptide to the infection media, and solutions were incubated at 37°C on a rotor wheel for 1 h to allow for viral infection.

Cells were then pelleted and resuspended in fresh medium at a concentration of 1×10^5 cells/mL. Each sample was added to wells on a tissue culture 96-well plate at a concentration of 10^4 cells in 100 μL of McCoy's 5A growth medium per well. They were incubated overnight at 37°C before infection was assessed using a luciferase assay as described in Myers et al.[36] A Quick Start Bradford protein assay (Bio-Rad, 5000201) was used to determine protein content in wells and adjust luminescence values for any differences.

In cell binding studies, the experimental setup was the same as that described for the second infection study, with 340,000 cells used per sample and a final concentration of 0.1 MOI. For blocked aMUC1-PCVV (MUC1-aMUC1-PCVV) samples MUC1 peptide was added to the infection media. After a 1-h incubation at 37°C on a rotor wheel, cells were pelleted and washed three times in 4 mL of PBS before being finally resuspended in 200 μL of PBS. Samples were DNA extracted using the GenElute mammalian genomic DNA miniprep kit protocol (Sigma-Aldrich, G1N350) and the quantity of VV genomes present was measured. Standards were made up in 340,000 CAPAN-2 cells and had a top concentration of 2.5×10^7 VG and decreased 1:10 to a lowest concentration of 25 VG in 5 μL. qPCR was performed on extracted DNA as in Myers et al.[36] Thermocycling parameters were 2 min at 95°C, followed by 40 cycles of 95°C (5 s) and 65°C (30 s).

In vivo *pharmacokinetic studies*

UK Home Office guidelines and the United Kingdom Co-ordinating Committee on Cancer Research (UKCCCR) Guidelines for the Welfare of Animals in Experimental Neoplasia were followed. All experiments were performed under a Home Office-approved project license by experimenters holding Home Office personal licenses. Female BALB/c nude mice at 5–7 weeks of age on arrival were used in all studies described herein. Mice were inoculated with a subcutaneous injection of 10^6 CAPAN-2 cells suspended in a total volume 100 μL of PBS, and dosing was performed when tumors reached a size of ~50–150 mm^3. The VV injectates were made up to a concentration of 1×10^6 or 5×10^6 VG/μL in a volume of 100 μL for each mouse. PCVV and aMUC1-PCVV were both made up as described in sections 4.1 and 4.2.3.1. VV, PCVV, and aMUC1-PCVV samples were incubated at 37°C for 1 h and was washed as described in section 4.1. Injectates were kept on ice until used.

Injection and blood collection

Mice were anesthetized with 2%–3% isoflurane in O_2-enhanced air prior to injection. Mice were bled at time points indicated on graphs by taking 20-40 μL from a tail vein puncture. All mice were sacrificed by cervical dislocation after 20 min. Livers and tumors were resected and stored at −80°C.

Sample processing

Blood samples, injectates, and homogenized tumors and livers (Precellys Evolution homogenizer, Bertin Instruments) were DNA extracted using the GenElute mammalian genomic DNA miniprep kit protocol (Sigma-Aldrich, G1N350), and the quantity of VV genomes present was measured. Standards were made up in mouse blood and had a top concentration of 2.5×10^7 VG and decreased 1:10 to a lowest concentration of 25 VG in 10 μL. DNA was isolated from blood samples and homogenates and then qPCR was performed as in Myers et al.[36] Thermocycling parameters were 2 min at 95°C, followed by 40 cycles of 95°C (5 s) and 65°C (30 s).

SUPPLEMENTAL INFORMATION

Supplemental information can be found online at https://doi.org/10.1016/j.omto.2021.03.011.

ACKNOWLEDGMENTS

The virus used in this study was generously supplied by Transgene SA, who also provided insights regarding the dosing and detection of VV and potential ligands for retargeting and provided edits to the manuscript. This work was supported by the Engineering and Physical Sciences Research Council (EPSRC) under Programme Grant EP/L024012/1 (OxCD3: Oxford Centre for Drug Delivery Devices). C.H was supported by a Linacre College Brewer Street Scholarship, M.G. was supported by a Clarendon Scholarship, and J.B. was supported by an All Souls Hugh Springer Scholarship.

AUTHOR CONTRIBUTIONS

C.H, M.G., L.B, S.W., J.B., and R.C. conceived, planned, and carried out the experiments. V.R synthesized the *O*-methyl-*O*′-[*N*-(cholesterylsuccinyl) aminoethyl]poly(ethylene glycol) 8 kDa, named within as Chol-PEG(8K) polymer. C.H. took the lead in writing the manuscript. All authors provided critical feedback and helped shape the research, analysis, and manuscript. C.C. helped supervise the project. V.R., K.F., and L.S. conceived the original idea. R.C. supervised the project.

DECLARATION OF INTERESTS

The authors declare no competing interests.

REFERENCES

1. Russell, S.J., Peng, K.-W., and Bell, J.C. (2012). Oncolytic virotherapy. Nat. Biotechnol. *30*, 658–670.
2. Hill, C., and Carlisle, R. (2019). Achieving systemic delivery of oncolytic viruses. Expert Opin. Drug Deliv. *16*, 607–620.
3. Chan, W.M., and McFadden, G. (2014). Oncolytic poxviruses. Annu. Rev. Virol. *1*, 119–141.
4. Hwang, T.-H., Moon, A., Burke, J., Ribas, A., Stephenson, J., Breitbach, C.J., Daneshmand, M., De Silva, N., Parato, K., Diallo, J.-S., et al. (2011). A mechanistic proof-of-concept clinical trial with JX-594, a targeted multi-mechanistic oncolytic poxvirus, in patients with metastatic melanoma. Mol. Ther. *19*, 1913–1922.
5. Mastrangelo, M.J., Maguire, H.C., Jr., Eisenlohr, L.C., Laughlin, C.E., Monken, C.E., McCue, P.A., Kovatich, A.J., and Lattime, E.C. (1999). Intratumoral recombinant GM-CSF-encoding virus as gene therapy in patients with cutaneous melanoma. Cancer Gene Ther. *6*, 409–422.
6. Breitbach, C.J., Moon, A., Burke, J., Hwang, T.H., and Kirn, D.H. (2015). A phase 2, open-label, randomized study of Pexa-Vec (JX-594) administered by intratumoral injection in patients with unresectable primary hepatocellular carcinoma. Methods Mol. Biol. *1317*, 343–357.
7. SillaJen, Inc. (2016). SillaJen announces first patient randomized in multinational phase 3 trial for Pexa-Vec in advanced liver cancer, https://www.prnewswire.com/news-releases/sillajen-announces-first-patient-randomized-in-multinational-phase-3-trial-for-pexa-vec-in-advanced-liver-cancer-300199554.html.
8. Heo, J., Reid, T., Ruo, L., Breitbach, C.J., Rose, S., Bloomston, M., Cho, M., Lim, H.Y., Chung, H.C., Kim, C.W., et al. (2013). Randomized dose-finding clinical trial of oncolytic immunotherapeutic vaccinia JX-594 in liver cancer. Nat. Med. *19*, 329–336.
9. Park, B.-H., Hwang, T., Liu, T.-C., Sze, D.Y., Kim, J.-S., Kwon, H.-C., Oh, S.Y., Han, S.-Y., Yoon, J.-H., Hong, S.-H., et al. (2008). Use of a targeted oncolytic poxvirus, JX-594, in patients with refractory primary or metastatic liver cancer: A phase I trial. Lancet Oncol. *9*, 533–542.
10. Kim, J.H., Oh, J.Y., Park, B.H., Lee, D.E., Kim, J.S.H.S., Park, H.E., Roh, M.S., Je, J.E., Yoon, J.H., Thorne, S.H., et al. (2006). Systemic armed oncolytic and immunologic therapy for cancer with JX-594, a targeted poxvirus expressing GM-CSF. Mol. Ther. *14*, 361–370.
11. Press release. (2012). Jennerex and Transgene present positive clinical data from phase 2 trial of JX594/TG6006 in sorafenib-refractory liver cancer patients. https://www.boursorama.com/bourse/actualites/jennerex-and-transgene-present-positive-clinical-data-from-phase-2-trial-of-jx594-tg6006-in-sorafenib-refractory-liver-cancer-patients-001b4bbde0eed99c0a4299270f367723.
12. Park, S.H., Breitbach, C.J., Lee, J., Park, J.O., Lim, H.Y., Kang, W.K., Moon, A., Mun, J.-H., Sommermann, E.M., Maruri Avidal, L., et al. (2015). Phase 1b trial of biweekly intravenous Pexa-Vec (JX-594), an oncolytic and immunotherapeutic vaccinia virus in colorectal cancer. Mol. Ther. *23*, 1532–1540.
13. Gaudin, R., and Barteneva, N.S. (2015). Sorting of small infectious virus particles by flow virometry reveals distinct infectivity profiles. Nat. Commun. *6*, 6022.
14. Cattaneo, R., Miest, T., Shashkova, E.V., and Barry, M.A. (2008). Reprogrammed viruses as cancer therapeutics: Targeted, armed and shielded. Nat. Rev. Microbiol. *6*, 529–540.
15. Hill, C.A.P., Bau, L., and Carlisle, R. (2020). Methods for modification of therapeutic viruses. Methods Mol. Biol. *2058*, 7–29.
16. Wonganan, P., and Croyle, M.A. (2010). PEGylated adenoviruses: From mice to monkeys. Viruses *2*, 468–502.
17. Fisher, K.D., Stallwood, Y., Green, N.K., Ulbrich, K., Mautner, V., and Seymour, L.W. (2001). Polymer-coated adenovirus permits efficient retargeting and evades neutralising antibodies. Gene Ther. *8*, 341–348.
18. Rojas, L.A., Condezo, G.N., Moreno, R., Fajardo, C.A., Arias-Badia, M., San Martín, C., and Alemany, R. (2016). Albumin-binding adenoviruses circumvent pre-existing neutralizing antibodies upon systemic delivery. J. Control. Release *237*, 78–88.
19. Mo, S., Carlisle, R., Laga, R., Myers, R., Graham, S., Cawood, R., Ulbrich, K., Seymour, L., and Coussios, C.-C. (2015). Increasing the density of nanomedicines improves their ultrasound-mediated delivery to tumours. J. Control. Release *210*, 10–18.
20. Morrison, J., Briggs, S.S., Green, N., Fisher, K., Subr, V., Ulbrich, K., Kehoe, S., and Seymour, L.W. (2008). Virotherapy of ovarian cancer with polymer-cloaked adenovirus retargeted to the epidermal growth factor receptor. Mol. Ther. *16*, 244–251.
21. Green, N.K., Morrison, J., Hale, S., Briggs, S.S., Stevenson, M., Subr, V., Ulbrich, K., Chandler, L., Mautner, V., Seymour, L.W., and Fisher, K.D. (2008). Retargeting polymer-coated adenovirus to the FGF receptor allows productive infection and mediates efficacy in a peritoneal model of human ovarian cancer. J. Gene Med. *10*, 280–289.
22. Hou, W., Sampath, P., Rojas, J.J., and Thorne, S.H. (2016). Oncolytic virus-mediated targeting of PGE_2 in the tumor alters the immune status and sensitizes established and resistant tumors to immunotherapy. Cancer Cell *30*, 108–119.
23. Thorne, S.H., Hwang, T.H.H., O'Gorman, W.E., Bartlett, D.L., Sei, S., Kanji, F., Brown, C., Werier, J., Cho, J.H., Lee, D.E., et al. (2007). Rational strain selection and engineering creates a broad-spectrum, systemically effective oncolytic poxvirus, JX-963. J. Clin. Invest. *117*, 3350–3358.
24. Rojas, J.J., Sampath, P., Bonilla, B., Ashley, A., Hou, W., Byrd, D., and Thorne, S.H. (2016). Manipulating TLR signaling increases the anti-tumor T cell response induced by viral cancer therapies. Cell Rep. *15*, 264–273.
25. Smith, G.L., and Moss, B. (1983). Infectious poxvirus vectors have capacity for at least 25 000 base pairs of foreign DNA. Gene *25*, 21–28.
26. Paul, S., Geist, M., Dott, K., Snary, D., Taylor-Papadimitriou, J., Acres, B., Silvestre, N., Kieny, M.-P., and Balloul, J.-M. (2007). Specific tumor cell targeting by a recombinant MVA expressing a functional single chain antibody on the surface of intracellular mature virus (IMV) particles. Viral Immunol. *20*, 664–671.
27. Scholl, S.M., Balloul, J.M., Le Goc, G., Bizouarne, N., Schatz, C., Kieny, M.P., von Mensdorff-Pouilly, S., Vincent-Salomon, A., Deneux, L., Tartour, E., et al. (2000). Recombinant vaccinia virus encoding human *MUC1* and *IL2* as immunotherapy in patients with breast cancer. J. Immunother. *23*, 570–580.
28. Taylor-Papadimitriou, J., Burchell, J., Miles, D.W., and Dalziel, M. (1999). MUC1 and cancer. Biochim. Biophys. Acta *1455*, 301–313.
29. Gendler, S.J. (2001). MUC1, the renaissance molecule. J. Mammary Gland Biol. Neoplasia *6*, 339–353.
30. Foloppe, J., Kempf, J., Futin, N., Kintz, J., Cordier, P., Pichon, C., Findeli, A., Vorburger, F., Quemeneur, E., and Erbs, P. (2019). The enhanced tumor specificity of TG6002, an armed oncolytic vaccinia virus deleted in two genes involved in nucleotide metabolism. Mol. Ther. Oncolytics *14*, 1–14.
31. Arakelyan, A., Fitzgerald, W., Margolis, L., and Grivel, J.-C. (2013). Nanoparticle-based flow virometry for the analysis of individual virions. J. Clin. Invest. *123*, 3716–3727.
32. Tang, V.A., Renner, T.M., Varette, O., Le Boeuf, F., Wang, J., Diallo, J.-S., Bell, J.C., and Langlois, M.-A. (2016). Single-particle characterization of oncolytic vaccinia virus by flow virometry. Vaccine *34*, 5082–5089.
33. Kurfürst, M.M. (1992). Detection and molecular weight determination of polyethylene glycol-modified hirudin by staining after sodium dodecyl sulfate-polyacrylamide gel electrophoresis. Anal. Biochem. *200*, 244–248.
34. Ercole, F., Whittaker, M.R., Quinn, J.F., and Davis, T.P. (2015). Cholesterol modified self-assemblies and their application to nanomedicine. Biomacromolecules *16*, 1886–1914.
35. Elsaid, N., Somavarapu, S., and Jackson, T.L. (2014). Cholesterol-poly(ethylene) glycol nanocarriers for the transscleral delivery of sirolimus. Exp. Eye Res. *121*, 121–129.
36. Myers, R., Coviello, C., Erbs, P., Foloppe, J., Rowe, C., Kwan, J., Crake, C., Finn, S., Jackson, E., Balloul, J.-M., et al. (2016). Polymeric cups for cavitation-mediated delivery of oncolytic vaccinia virus. Mol. Ther. *24*, 1627–1633.
37. Carlisle, R., Choi, J., Bazan-Peregrino, M., Laga, R., Subr, V., Kostka, L., Ulbrich, K., Coussios, C.-C., and Seymour, L.W. (2013). Enhanced tumor uptake and penetration of virotherapy using polymer stealthing and focused ultrasound. J. Natl. Cancer Inst. *105*, 1701–1710.
38. Kyriazis, A.A., Kyriazis, A.P., Sternberg, C.N., Sloane, N.H., and Loveless, J.D. (1986). Morphological, biological, biochemical, and karyotypic characteristics of human

pancreatic ductal adenocarcinoma Capan-2 in tissue culture and the nude mouse. Cancer Res. *46*, 5810–5815.

39. Fisher, K.D., Green, N.K., Hale, A., Subr, V., Ulbrich, K., and Seymour, L.W. (2007). Passive tumour targeting of polymer-coated adenovirus for cancer gene therapy. J. Drug Target. *15*, 546–551.

40. Carlisle, R.C., Di, Y., Cerny, A.M., Sonnen, A.F.-P.P., Sim, R.B., Green, N.K., Subr, V., Ulbrich, K., Gilbert, R.J.C., Fisher, K.D., et al. (2009). Human erythrocytes bind and inactivate type 5 adenovirus by presenting Coxsackie virus-adenovirus receptor and complement receptor 1. Blood *113*, 1909–1918.

41. Green, N.K., Herbert, C.W., Hale, S.J., Hale, A.B., Mautner, V., Harkins, R., Hermiston, T., Ulbrich, K., Fisher, K.D., and Seymour, L.W. (2004). Extended plasma circulation time and decreased toxicity of polymer-coated adenovirus. Gene Ther. *11*, 1256–1263.

42. Green, N.K., Hale, A., Cawood, R., Illingworth, S., Herbert, C., Hermiston, T., Subr, V., Ulbrich, K., van Rooijen, N., Seymour, L.W., and Fisher, K.D. (2012). Tropism ablation and stealthing of oncolytic adenovirus enhances systemic delivery to tumors and improves virotherapy of cancer. Nanomedicine (Lond.) *7*, 1683–1695.

43. Evgin, L., Acuna, S.A., Tanese de Souza, C., Marguerie, M., Lemay, C.G., Ilkow, C.S., Findlay, C.S., Falls, T., Parato, K.A., Hanwell, D., et al. (2015). Complement inhibition prevents oncolytic vaccinia virus neutralization in immune humans and cynomolgus macaques. Mol. Ther. *23*, 1066–1076.

44. Akin, D., Li, H., and Bashir, R. (2004). Real-time virus trapping and fluorescent imaging in microfluidic devices. Nano Lett. *4*, 257–259.

45. Tomita, U., Yamaguchi, S., Sugimoto, Y., Takamori, S., and Nagamune, T. (2012). Poly(ethylene glycol)-lipid-conjugated antibodies enhance dendritic cell phagocytosis of apoptotic cancer cells. Pharmaceuticals (Basel) *5*, 405–416.

OMTO, Volume *21*

Supplemental information

Polymer stealthing and mucin-1 retargeting for enhanced pharmacokinetics of an oncolytic vaccinia virus

Claudia Hill, Megan Grundy, Luca Bau, Sheena Wallington, Joel Balkaran, Victor Ramos, Kerry Fisher, Len Seymour, Constantin Coussios, and Robert Carlisle

Polymer Stealthing and Mucin-1 (MUC1) Retargeting for Enhanced Pharmacokinetics of an Oncolytic Vaccinia Virus

Claudia Hill[1], Megan Grundy[1], Luca Bau[1], Sheena Wallington[1], Joel Balkaran[1], Victor Ramos[2], Kerry Fisher[3], Len Seymour[3], Constantin Coussios[1] and Robert Carlisle[1]

[1] Institute of Biomedical Engineering, University of Oxford, UK, [2] Grup d'Enginyeria de Materials, Institut Quimic de Sarria, Universitat Ramos Llull, [3] Department of Oncology, University of Oxford, UK

This work was performed in Oxford, UK. Correspondence should be addressed to R.C. (robert.carlise@eng.ox.ac.uk)

Short title: Polymer coating and retargeting of oncolytic VV

Keywords: vaccinia virus; oncolytic virotherapy; polymer coating; polymer coating; cholesterol-PEG; targeting; mucin 1; capan-2; pancreatic cancer

Supplementary information:

Methods:

In a further biodistribution study, mice were dosed with $1x10^8$ VG of luciferase expressing VV, PCVV or aMUC1-PCVV (Figure S3). 24 hours after mice were treated with $1x10^8$ VG IVIS imaging was performed to assess the biodistribution and luminescence in livers and tumours. Mice were anaesthetised with 2-3% isoflurane in O2 enhanced air and then were injected intraperitoneally with 100µL of 15.8 mg/mL luciferin (15225733, ThermoFisher). 100µL Pierce™ D-Luciferin, Monopotassium Salt (ThermoFisher Scientific, 88294) was used at 15.8 mg/mL in PBS by intraperitoneal injection. The dose was split between two injections of 50µL on each side of the abdomen of the mouse being imaged. 5 minutes after luciferin administration, imaging was performed using an in vivo imaging system (IVIS Lumina, Caliper Life Sciences).

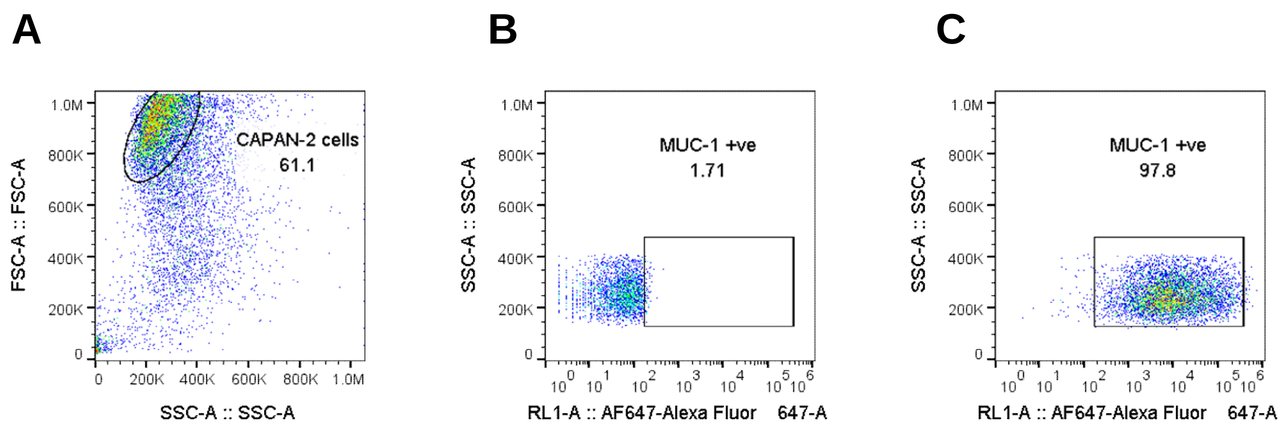

Figure S1 Characterising the binding of aMUC-1 antibodies to a high MUC-1 expressing human cancer cell line CAPAN-2. Flow cytometry was used to determine the binding of aMUC-1 antibodies to CAPAN-2 cells. Cells were harvested and suspended at a $1x10^3$ cells per mL concentration and groups that were stained for MUC-1 were incubated with a 40 µg/ mL concentration of aMUC-1 antibody. Cells were then washed in PBS and aMUC-1 binding was detected using an anti-mouse IgG AF647 secondary. (a) Gating for CAPAN-2 cell population. (b) Gating for positive MUC-1 staining set by unstained CAPAN-2 cells and (c) CAPAN-2 cells stained for MUC-1.

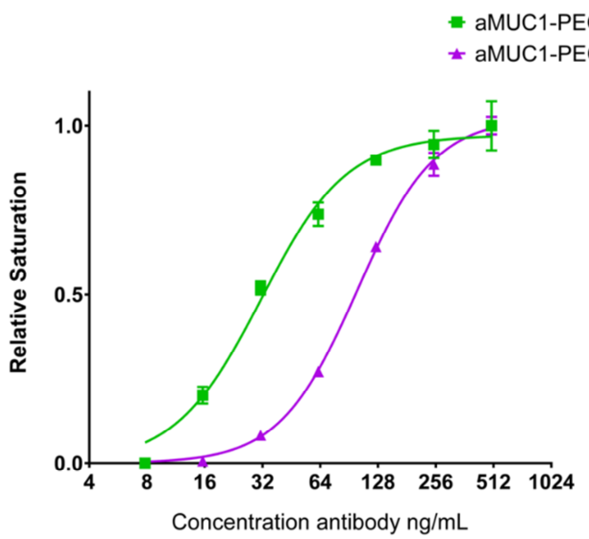

Figure S2 Assessing the impact of incubation of MUC1 peptide on Cholesterol-PEG-aMUC1 binding to immobilised MUC1. An ELISA was performed to compare the binding affinity of aMUC1-PEG-Chol suspended at different concentrations in PBS (green) and aMUC1-PEG-Chol incubated suspended at different concentrations in PBS containing five times the mass of aMUC1 of free MUC1 core peptide (purple) to immobilised MUC1 core peptide on the plate, using an ELISA. Error bars represent SD, n=5.

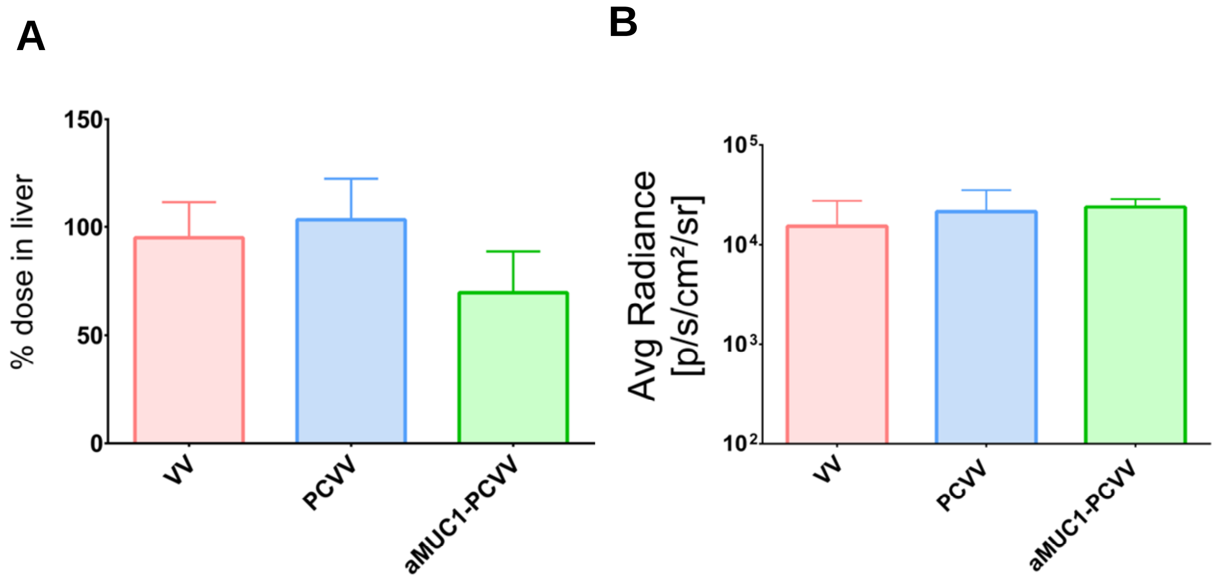

Figure S3 Analysis of livers after dosing with 1x10^8 VG of VV (pink), PCVV (blue) and aMUC1-PCVV (green). (A) Livers were excised from mice 20 minutes after dosing in PK studies and the % dose of VV, PCVV or aMUC1-PCVV circulating was determined by qPCR for each liver. (B) The mean average radiance was determined in livers 24 hours after dosing. No significant differences were evident between any group in panel A or B. n=4
